# AHR‐Responsive Carbon Dots Orchestrate Skin Wound Healing and Colonic Mucosal Repair via Treg‐Mediated Macrophage Efferocytosis

**DOI:** 10.1002/advs.77526

**Published:** 2026-09-06

**Authors:** Zilu Zhu, Han Zhang, Shuai Lin, Wenqian Zheng, Xin Li, Xiaowei Xu, Ruili Yang

**Affiliations:** ^1^ Department of Orthodontics National Center for Stomatology & National Clinical Research Center for Oral Diseases & National Engineering Research Center of Oral Biomaterials and Digital Medical Devices Beijing Key Laboratory of Digital Stomatology & NHC Key Laboratory of Digital Stomatology & NMPA Key Laboratory for Dental Materials Beijing Peking University School and Hospital of Stomatology China; ^2^ Jilin Provincial Key Laboratory of Tooth Development and Bone Remodeling Hospital of Stomatology Jilin University Changchun China; ^3^ Division of Restorative Dental Sciences Faculty of Dentistry The University of Hong Kong Hong Kong China; ^4^ Department of Stomatology The First Hospital of Jilin University Jilin University Changchun China

**Keywords:** aryl hydrocarbon receptor, carbon dots, CCL1, gut–skin axis, macrophage efferocytosis, treg cells

## Abstract

As critical interface organs, the gut and skin share substantial immunological similarities, and gut diseases are often accompanied by skin comorbidities, yet the underlying mechanisms remain unclear. Here, we observed delayed skin wound healing in a mouse model of colitis. To promote both skin and intestinal repair, we designed aryl hydrocarbon receptor (AHR)–responsive carbon dots using pharmacophore modeling and fragment‐based drug design. These nanoscale carbon dots significantly alleviated colitis and accelerated impaired skin wound healing. The carbon dots preferentially accumulated in the inflamed colon, where they induced infiltration of tissue‐resident regulatory T (Treg) cells, thereby suppressing mucosal inflammation and promoting tissue repair. Mechanistically, the carbon dots activated AHR signaling to drive Treg cell differentiation. Importantly, Treg‐derived chemokine CCL1 enhanced macrophage efferocytosis, establishing a pro‐regenerative immune microenvironment in both the colon and skin wounds via STAT3–SCARB1 signaling. Notably, *Ccl1* knockdown attenuated the therapeutic effects of carbon dots on both colonic mucosal repair and skin wound healing, supporting CCL1 as a key mediator of gut–skin immune communication. Collectively, this study highlights the important role of Treg cells in gut–skin axis crosstalk and provides a nanomaterial‐based strategy for coordinated tissue repair.

## Graphical abstract

Guided by pharmacophore modeling and molecular docking, aryl hydrocarbon receptor (AHR)‐responsive carbon dots were synthesized. They bind directly to AHR and expand gut‐resident regulatory T (Treg) cells. Treg‐derived C‐C motif chemokine ligand 1 (CCL1) enhances macrophage efferocytosis, creating a pro‐regenetative niche that promotes colonic mucosal repair and skin wound healing via the gut‐skin axis. Created with BioRender.

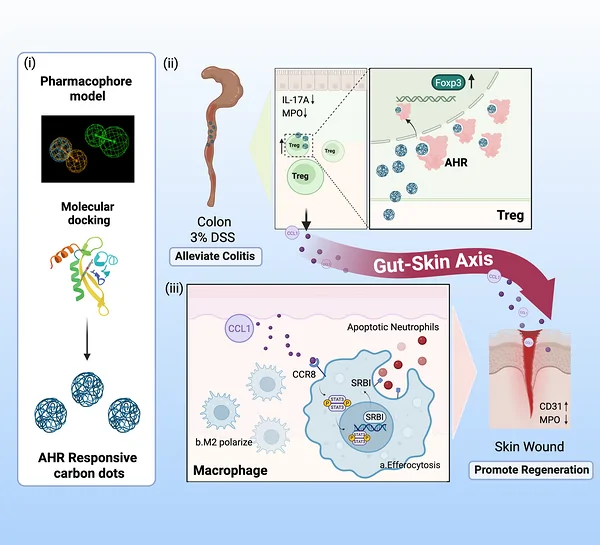

## 1 Introduction

Inflammatory bowel disease (IBD), including ulcerative colitis (UC) and Crohn's disease (CD), is a chronic and relapsing inflammatory disorder and has become an increasing global health concern [1, 2]. Beyond its gastrointestinal impacts, IBD is often accompanied by extraintestinal manifestations. Approximately 15–20% of IBD patients frequently present with skin conditions, including skin ulcers, vasculitis, hair loss, erythema nodosum, and pyoderma gangrenosum [3]. In recent years, growing evidence has revealed cellular crosstalk between the gut and skin, a concept referred to as the “gut–skin axis” [4]. Both the gut and skin are lined by epithelial cells, extensively vascularized and innervated. These structural features allow them to communicate rapidly with the immune system, thereby facilitating quick responses to environmental threats [7, 8, 9, 10]. However, the mechanisms coordinating immune responses between the gut and skin remain incompletely understood.

Microbiota‐derived metabolites have emerged as important mediators of gut–skin communication. Short‐chain fatty acids (SCFAs), including acetate, propionate, and butyrate, are generated by bacterial fermentation of dietary fibers and have been shown to regulate epidermal barrier integrity by promoting keratinocyte mitochondrial metabolism and differentiation [5, 6]. In addition, bile acids represent another class of host–microbial co‐metabolites involved in systemic immune regulation and skin inflammation. Secondary bile acids derived from intestinal microbial metabolism can attenuate psoriasiform dermatitis by suppressing IL‐17A production and CCL20–CCR6‐mediated T‐cell trafficking. Conversely, dysregulated bile acid metabolism has also been associated with keratinocyte metabolic dysfunction and inflammatory skin diseases [7, 8]. These findings raise the possibility that modulating gut‐derived immune signals may provide a strategy to coordinate intestinal and skin wound repair.

The aryl hydrocarbon receptor (AHR), a ligand‐dependent transcription factor and immune sensor, is critically involved in the pathogenesis of IBDs [9, 10]. AHR is expressed in multiple immune‐cell populations and participates in diverse immune responses [11, 12]. It drives the development of gut innate lymphoid cells, providing a potential target for intestinal disorders treatment [13, 14]. Moreover, AHR regulates regulatory T (Treg) and Th17 cell differentiation in a ligand‐specific manner, with distinct ligands activating AHR to exert different effects. For instance, activation of AHR by 2,3,7,8‐tetrachlorodibenzo‐p‐dioxin (TCDD) induces functional Treg cells that suppress experimental autoimmune encephalomyelitis (EAE). In contrast, activation of AHR by 6‐formylindolo[3,2‐b]carbazole (FICZ) interferes with Treg cell development, promotes Th17 cell differentiation, and exacerbates EAE severity in mice [15]. Recent studies have also highlighted the role of dietary tryptophan metabolites in maintaining gut homeostasis by binding and activating AHR. For example, indole‐3‐acetic acid (IAA) alleviates dextran sulfate sodium (DSS)‐induced colitis by enhancing equol production through *Bifidobacterium pseudolongum* [16]. Additionally, *Bifidobacterium longum* improves atopic dermatitis by regulating tryptophan metabolism [17]. Notably, AHR‐deficient mice exhibit increased susceptibility to DSS‐induced colitis, underscoring its critical role in immune homeostasis [18, 19]. These findings suggest that AHR may be a potential target for mediating gut–skin crosstalk. However, the mechanism by which AHR regulates gut–skin crosstalk is still largely unknown.

Carbon dots, small carbon nanoparticles less than 10 nm in size, exhibit excellent biocompatibility. Carbon dots are mainly composed of carbon, an abundant and relatively biocompatible element that is also a fundamental component of biological molecules. Moreover, their biodistribution and surface functional groups can be tuned through precursor selection and synthesis conditions [20, 21]. However, whether engineered functional groups in carbon dots can regulate AHR signaling remains unclear.

In this study, we designed AHR‐responsive carbon dots using pharmacophore modeling and fragment‐based drug design, which directly bind to AHR and promote the expansion of Treg cells. Notably, CCL1 secreted by these Treg cells enhanced macrophage efferocytosis, thereby establishing a reparative microenvironment that accelerated both skin wound healing and colonic mucosal repair. Together, this study presents a nanomedicine strategy to modulate the gut–skin axis, offering a potential approach for treating coordinated barrier injuries in both mucosal and skin tissues.

## 2 Results

### 2.1 Altered AHR Expression in CD4^+^ T Cells From Patients With IBD

**FIGURE 1:**
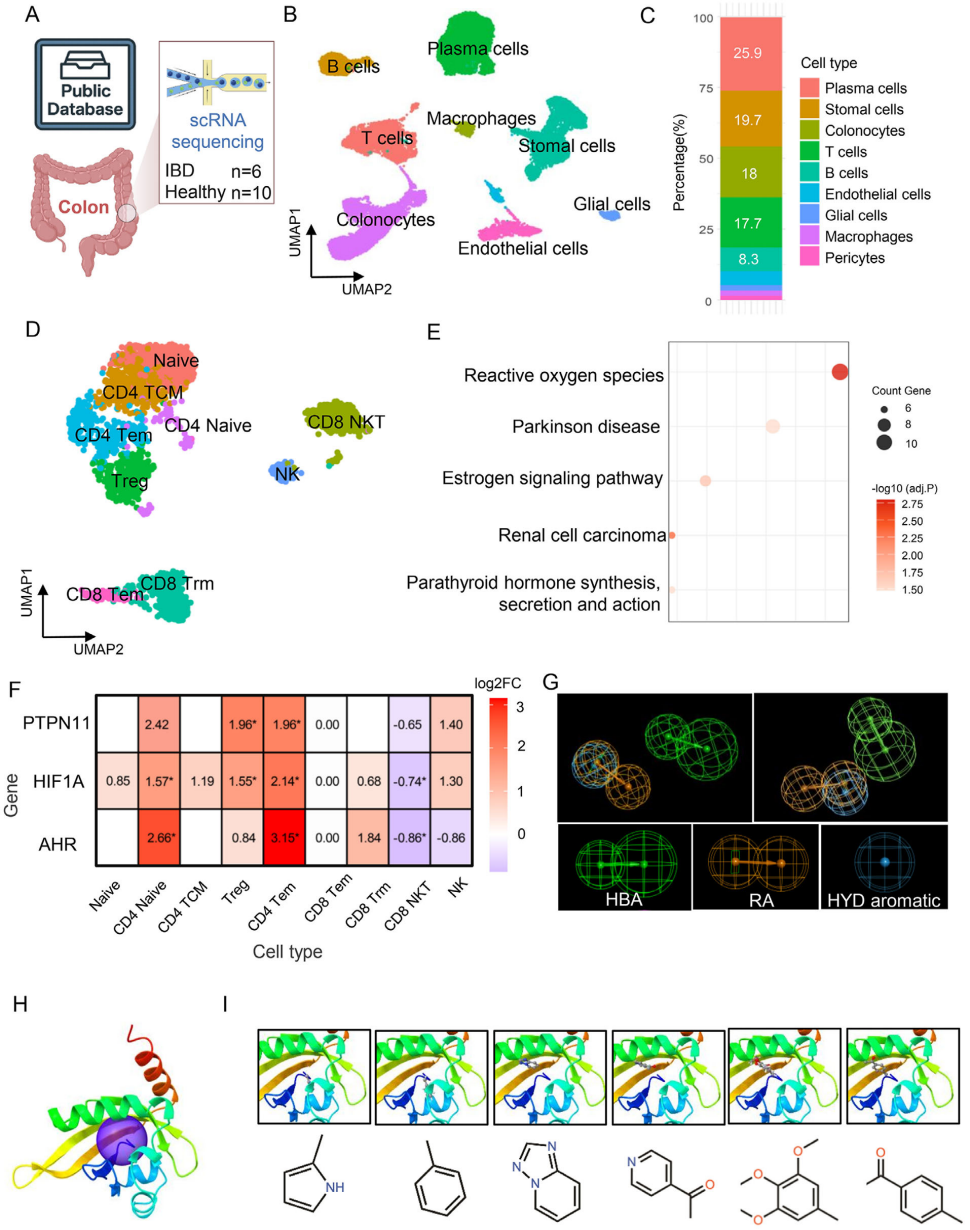
Integrated single‐cell transcriptomic analysis and AHR ligand design in colonic T cell populations of IBD. (A) Schematic workflow. This schematic was created by BioRender. (B) UMAP visualization of cells derived from colon tissues of control and IBD patients, including T cells, B cells, macrophages, plasma cells, stromal cells, pericytes, glial cells, colonocytes, and endothelial cells. (C) Stacked bar plot showing the composition of major cell types across IBD and control samples. (D) UMAP visualization of the populations of a total 48, 091 T cells and NK cells, including naïve T cells, CD4^+^ naïve T cells, regulatory T cells, CD4^+^ effector memory T cells, CD4^+^ central memory T cells, CD8^+^ effector memory T cells, CD8^+^ tissue‐resident memory T cells, CD8^+^ natural killer T‐like cells, and natural killer cells. (E) KEGG pathway enrichment analysis of differentially expressed genes in T cell populations from IBD and control colon tissues. (F) Heatmap displaying the log2 fold changes and p‐values for the expression of PTPN11, HIF1A, and *AHR* across T cell subpopulations. (G) Pharmacophore model showing key interaction features of AHR ligands, hydrogen bond acceptor (HBA, green), ring aromatic (RA, orange), and hydrophobic aromatic (HYD, blue). (H) Simulated 3D structure of the AHR‐ligand complex, illustrating multiple binding interfaces. (I) Six functional groups of AHR ligands predicted by fragment‐based drug design. These chemical structures were created using ChemDraw. Naive, Naïve T cells. CD4 Naive, CD4^+^ naïve T cells. Treg, regulatory T cells. CD4^+^ Tem, CD4^+^ effector memory T cells. CD4^+^ Tcm, CD4^+^ central memory T cells. CD8^+^ Tem, CD8^+^ effector memory T cells. CD8^+^ Trm, CD8^+^ tissue‐resident memory T cells. CD8 NKT, CD8^+^ natural killer T‐like cells. NK, natural killer cells. *p < 0.05, **p < 0.01, ***p < 0.001.

To systematically investigate the cellular composition and diversity in IBD patients, we integrated single‐cell RNA sequencing data from colonic tissues across four public datasets [22, 23, 24] using the Harmony algorithm, yielding a comprehensive IBD‐related single‐cell atlas comprising 36,056 cells from 16 samples (22,277 control cells and 13,779 IBD cells, Figures 1A and S1A). Principal component analysis (PCA) of highly variable genes, followed by clustering, identified nine major cell types (Figures 1B and S1B). T cells are among the most abundant immune cell populations in the colon (Figure 1C), and T cell dysregulation is closely linked to the pathogenesis of IBD [25]. Thus, T cells were further divided into nine subpopulations as follows: naïve T cells (Naive), CD4^+^ naïve T cells (CD4 Naive), CD4^+^ central memory T cells (CD4 Tcm), regulatory T cells (Treg), CD4^+^ effector memory T cells (CD4^+^ Tem), CD8^+^ effector memory T cells (CD8^+^ Tem), CD8^+^ tissue‐resident memory T cells (CD8^+^ Trm), CD8^+^ natural killer T‐like cells (CD8 NKT), and natural killer cells (NK) (Figures 1D and S1C).

We then performed differential expression analysis between the IBD and control groups, followed by Kyoto Encyclopedia of Genes and Genomes (KEGG) pathway enrichment. The reactive oxygen species pathway emerged as the most significantly upregulated pathway (Figure 1E). To explore the involvement of this pathway in specific immune cell populations, we projected the gene set onto T‐cell populations. This analysis revealed distinct expression patterns of *AHR*, *HIF1A*, and *PTPN11* across different T cell populations (Figure S1D,E). Notably, the upregulation of AHR and HIF1A was predominantly observed in CD4^+^ T‐cell subsets (CD4^+^Naïve, CD4^+^ Tem) (Figure 1F). Multiple AHR agonists, such as indole‐3‐carbinol and FICZ, have been shown to significantly ameliorate colitis in mouse models [26, 27]. Furthermore, elevated AHR expression was detected in CD4^+^ T cells of DSS‐induced colitis mice (Figure S1F). These results imply the potential of AHR as a therapeutic target in IBD.

### 2.2 Synthesis and Characterization of AHR‐Responsive Carbon Dots

**FIGURE 2:**
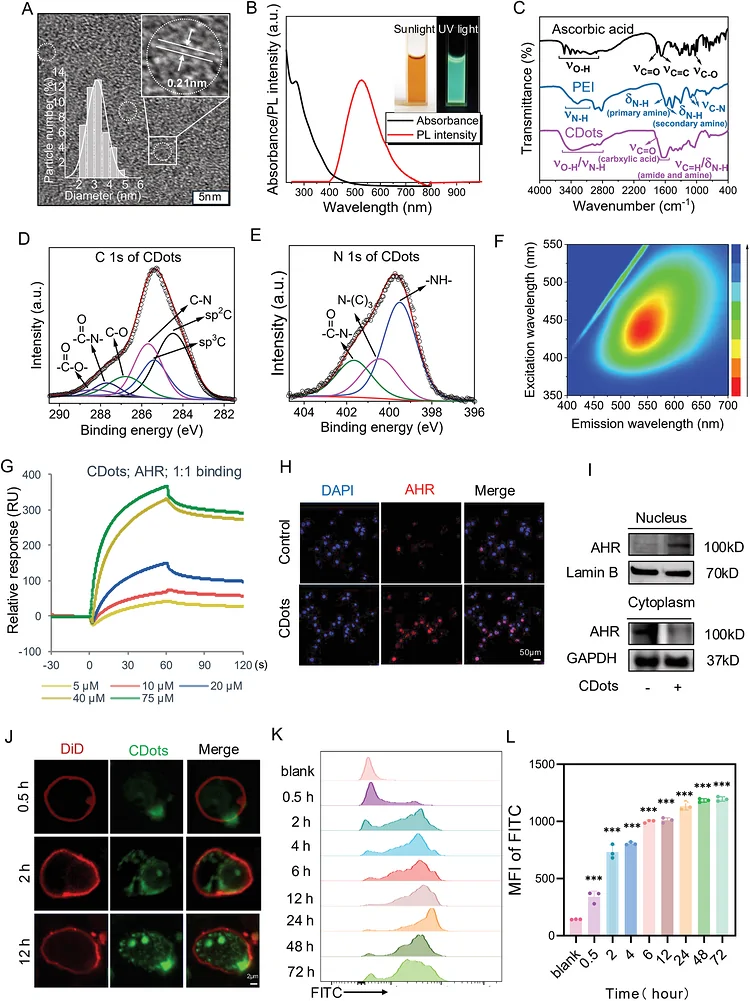
Physicochemical characterization of AHR‐responsive carbon dots. (A) The crystal structure of carbon dots analyzed by high‐resolution transmission electron microscopy (HRTEM). (B) Ultraviolet‐Visible absorption spectra (black) and photoluminescence (PL) spectra (red, λ_ex_  =  405 nm) with photographic insets under sunlight (left) and UV light (right) for carbon dots. (C) Fourier‐transform infrared spectroscopy spectra of ascorbic acid (black), PEI (blue), and carbon dots (red), confirming surface functionalization. (D, E) High‐resolution XPS spectra for C 1s and N 1s peaks of carbon dots. (F) Excitation‐emission matrix profile of carbon dots. (G) The binding affinity of carbon dots to AHR as assessed by surface plasmon resonance (SPR) analysis. (H) The expression of AHR in control and carbon dot‐treated T cells analyzed by immunofluorescence staining (I) The expression of AHR in control and carbon dot‐treated T cell cytoplasm and nucleus, respectively. (J) The endocytosis of carbon dots (green) by T cells (red) after coculture for 0.5, 2 and 12 h. (K, L) The representative flow cytometry images and quantification of carbon dots uptake by T cells, as assessed by flow cytometry. Data are presented as mean ± SD. CDots, carbon dots. *p < 0.05, **p < 0.01, ***p < 0.001.

To design the AHR‐responsive carbon dots, we first analyzed the features required for AHR recognition. Pharmacophore modelling identified three key interaction features: hydrogen bond acceptor sites, aromatic ring sites, and hydrophobic/aromatic sites (Figure 1G). Furthermore, fragment‐based drug design was performed based on the AHR protein model (UniProt ID: P35869) (Figure 1H). Six representative AHR antagonists (Figure S2A) and ten AHR agonists (Figure S2B) were docked into the receptor, revealing the typical functional group preferences for AHR ligands, including nitrogen‐containing and oxygen‐containing aromatic rings (Figure 1I). Guided by these models, we next synthesized AHR‐responsive carbon dots via a one‐step microwave‐assisted reaction using ascorbic acid and polyethyleneimine as precursors (Figure S3A). High‐resolution transmission electron microscopy images revealed a uniform particle size (average diameter ∼3.5 nm) with a lattice spacing of 0.21 nm, consistent with the characteristic interlayer distance of graphitic carbon (Figure 2A). Under UV light, the carbon dot solution but not its precursor solutions exhibited a bright green emission, while appearing pale yellow under sunlight (Figures 2B and S3B,C). Spectroscopic analysis verified the retention of key functional groups after carbonization. Fourier transform infrared spectroscopy spectra of carbon dots displayed characteristic C═O stretching (1720 cm^−^^1^), a broad O‐H/N‐H band, and an amide‐related peak near 1592 cm^−^^1^, indicating the presence of hydroxyl, carboxyl, and amino groups (Figure 2C). X‐ray photoelectron spectroscopy (XPS) further supported this, with C 1s peaks assigned to sp^2^ carbon, sp^3^ carbon, C‐N, C‐O, ‐CON‐, and ‐COO^−^ species, and N 1s peaks corresponding to ‐NH‐, graphitic nitrogen, and amide nitrogen (Figure 2D,E). The optical characterization showed that carbon dots emitted strong green fluorescence (Figure 2F). Zeta potential analysis showed that the carbon dots carried a negative surface charge, with a zeta potential of −9.529 mV (Figure S3D).

Next, we analyzed the interaction between carbon dots and AHR. A simplified carbon dot fragment was used to mimic carbon dots, and the 120 ns molecular dynamics trajectory demonstrated a stable binding conformation within the AHR ligand‐binding pocket (Figure S3E‐G). Root‐mean‐square deviation (RMSD) analysis indicated that the AHR‐carbon dot complex reached equilibrium after ∼90 ns, with RMSD stabilizing at around 2.5 Å (Figure S3H). Secondary structure analysis confirmed that the overall fold of AHR, including α‐helices and β‐sheets, remained stable throughout the simulation (Figure S3I). Notably, root‐mean‐square fluctuation (RMSF) analysis revealed reduced flexibility of residues within the binding pocket upon carbon dots binding, whereas ligand‐free AHR exhibited increased mobility in this region (Figure S3J), indicating that carbon dots directly engaged the ligand‐binding domain and stabilized its conformational dynamics. Furthermore, surface plasmon resonance (SPR) analysis verified the direct interaction between AHR and carbon dots with a dissociation constant (KD) of 2.35 µM (Figure 2G). These characteristics imply that carbon dots directly interact with AHR.

Functionally, carbon dot treatment promoted AHR nuclear translocation in T cells, as shown by immunofluorescence staining and western blotting (Figures 2H,I and S4A). Consistently, the expression of AHR downstream target genes, including *Ahrr*, *Cyp1a1*, and *Cyp1b1* [28], was increased after carbon dot treatment (Figure S4B–D). Furthermore, carbon dots were internalized by T cells in a time‐dependent manner (Figure 2J–L). These results indicate that carbon dots are taken up by T cells and activate AHR signaling.

### 2.3 AHR‐Responsive Carbon Dots Promoted Treg Cell Polarization

We first evaluated the in vitro biosafety of carbon dots to identify an appropriate working concentration. The results demonstrated that carbon dot treatment did not induce T cell apoptosis at concentrations up to 400 µg mL^−1^. Furthermore, carbon dots improved T cell viability at concentrations ranging from 100 to 200 µg mL^−1^. However, T cell viability declined when the carbon dot concentration reached 400 µg mL^−1^ (Figure S4E,F). On the basis of these results, 200 µg mL^−1^ was selected for subsequent T‐cell polarization assays.

**FIGURE 3:**
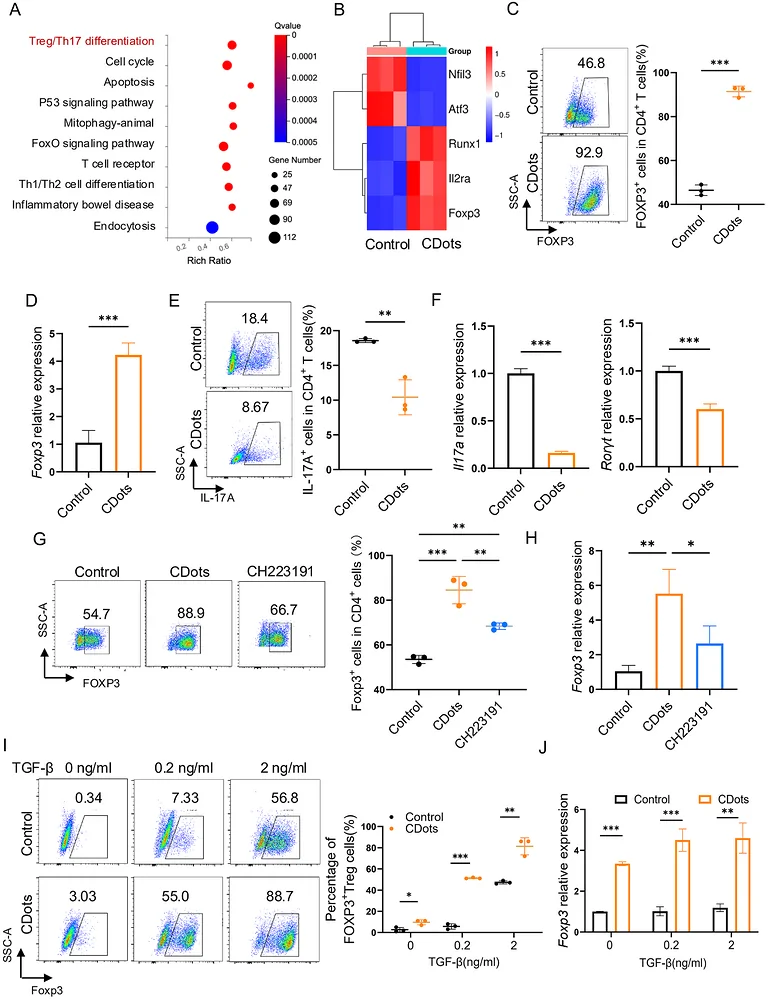
AHR‐responsive carbon dots promoted Treg cell polarization. (A) The top 10 significantly enriched signaling pathways based on the differentially expressed genes between control and carbon dot‐treated T cells analyzed by KEGG. Treg/Th17 differentiation related pathway was the most significant one. (B) The significantly different genes related with Treg/Th17 differentiation between control and carbon dot‐treated groups. (C) FOXP3^+^ Treg cells ratio in CD4^+^ T cells between control and carbon dot‐treated groups in the Treg cell polarization induction condition, as assessed by flow cytometry. (D) The gene expression of *Foxp3* in control and carbon dot‐treated CD4^+^ T cells under Treg cell polarization induction condition analyzed by RT‐qPCR. (E) IL‐17A positive cells ratio in CD4^+^ T cells between control and carbon dot‐treated groups under Th17 cell polarization induction condition, as assessed by flow cytometry. (F) The gene expression of *Il17a* and *Rorγt* in control and carbon dot‐treated CD4^+^ T cells under Th17 cell polarization induction condition, analyzed by RT‐qPCR. (G, H) The ratio of FOXP3^+^ Treg cells (G) and gene expression of *Foxp3* (H) in control and carbon dot treated groups with or without AHR antagonist CH223191 treatment. (I, J) The ratio of FOXP3^+^ Treg cells (I) and gene expression of *Foxp3* (J) treated by different concentrations of TGF‐β (0, 0.2 and 2 ng/mL) with or without carbon dots. Data are presented as mean ± SD. CDots, carbon dots. *p < 0.05, **p < 0.01, ***p < 0.001.

To examine how AHR‐responsive carbon dots affect T cells, we compared the gene profiles of control and carbon dot‐treated T cells using RNA‐sequencing analysis (Figure S5A). Enrichment of Treg/Th17 differentiation pathway after carbon dot treatment was detected, as assessed by KEGG (Figure 3A). Moreover, the levels of *Foxp3* and *Runx1* were significantly increased after carbon dot treatment (Figure 3B). Next, we analyzed whether carbon dots regulated T cell polarization. The results revealed that Treg cell polarization was significantly increased after carbon dot treatment, accompanied with increased expression of master transcription factor *Foxp3* (Figure 3C,D). The differentiation of Th17 cells was also inhibited by carbon dot treatment, accompanied by the decreased expression of *Il17a* and *RORγt* (Figure 3E,F). Carbon dot treatment did not alter the differentiation of Th1 cells or T cell proliferation (Figure S5B‐D). To determine the role of AHR in T cell subsets polarization, AHR antagonist CH223191 was employed to treat T cells. The results revealed that the increased proportion of Treg cells induced by carbon dot treatment were substantially inhibited by CH223191 administration (Figure 3G,H). While CH223191 treatment failed to alter Th17 differentiation (Figure S5E). These results indicated that AHR signaling is essential for carbon dot‐induced Treg cell differentiation. Moreover, Treg cell polarization induced by carbon dots was enhanced in the presence of TGF‐β in a dose‐dependent manner (Figure 3I,J). These results implied that carbon dots could promote Treg cell differentiation in an AHR‐dependent manner.

### 2.4 AHR‐Responsive Carbon Dots Alleviated DSS‐Induced Ulcerative Colitis

We next evaluated the therapeutic effects of carbon dots in vivo using a DSS‐induced colitis model in C57BL/6J mice. To determine an appropriate therapeutic dose, mice with DSS‐induced colitis were treated with carbon dots at 10, 20, or 40 mg kg^−1^. DSS administration resulted in marked colon shortening, progressive body weight loss, increased disease activity index (DAI) scores, and severe disruption of colonic architecture. Carbon dots at 20 and 40 mg kg^−1^ significantly improved colon length, body weight and colonic histology, decreased DAI scores,  whereas no significant improvement was observed at 10 mg kg^−1^ (Figure S6A‐F).

We further evaluated the systemic biosafety of carbon dots. Routine hematological parameters, including white blood cell count, red blood cell count, granulocyte count, lymphocyte count, platelet count, and hemoglobin, showed no obvious abnormalities following carbon dot administration (Figure S7A–F). Similarly, serum biochemical parameters, including alanine aminotransferase (ALT), aspartate aminotransferase (AST), creatinine (CRE), and blood urea nitrogen (BUN), remained comparable among the different groups (Figure S7G–J). H&E staining of the liver, lung, kidney, heart, and spleen revealed no evident pathological alterations after treatment with carbon dots at 10, 20, or 40 mg kg^−1^ (Figure S7K). Since 20 mg kg^−1^ carbon dots treatment revealed significant therapeutic efficacy with no detectable systemic toxicity, this dose was chosen for subsequent in vivo experiments.

**FIGURE 4:**
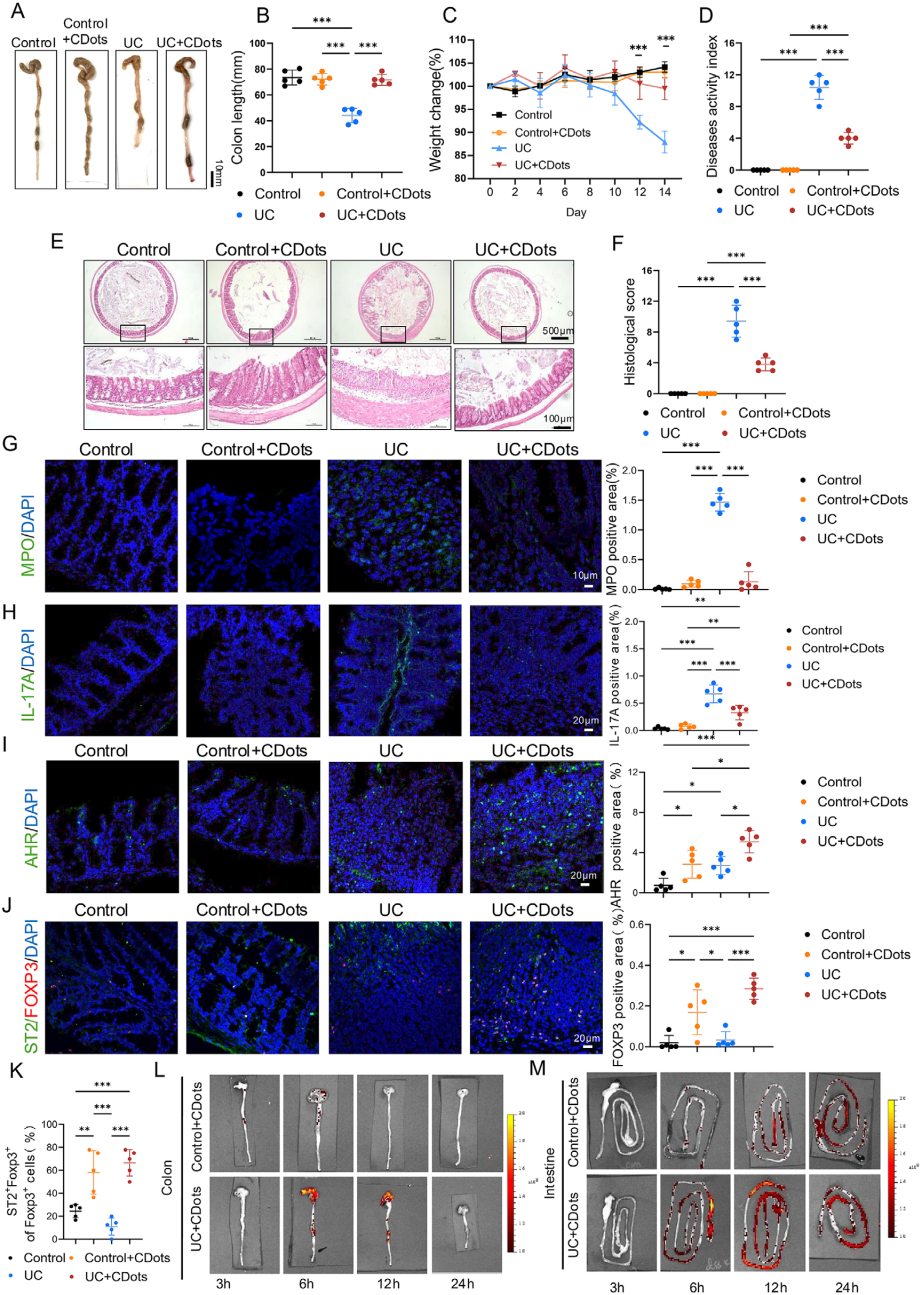
AHR‐responsive carbon dots alleviated DSS‐induced colitis in mice. (A) Representative images of colon of control and DSS‐induced colitis mice with or without carbon dot treatment. (B) The quantification of colon length in control and DSS‐induced colitis mice with or without carbon dot treatment. (C) The body weight change in control and DSS‐induced colitis mice with or without carbon dot treatment. (D) The disease activity index (DAI) scores of controls and DSS‐induced colitis mice with or without carbon dot treatment. (E) The histological structure of colons in control and DSS‐induced colitis mice with or without carbon dot treatment, as assessed by H&E staining. (F) The histological scores in control and DSS‐induced colitis mice with or without carbon dot treatment. (G‐I) The expression and quantification of MPO (G), IL‐17A (H), and AHR (I) in the colon of control and DSS‐induced colitis mice with or without carbon dot treatment, as assessed by immunofluorescence staining. (J, K) The expression and quantification of FOXP3 and ST2 in the colon of control and DSS‐induced colitis mice with or without carbon dot treatment, as assessed by immunofluorescence staining. (L, M) The accumulation of carbon dots in colon (L) and intestine (M) of control and DSS‐induced colitis mice at 3, 6, 12, and 24 h after carbon dot injection. Data are presented as mean ± SD. n = 5 mice per group. CDots, carbon dots. UC, DSS‐induced colitis. MPO, Myeloperoxidase. *p < 0.05, **p < 0.01, ***p < 0.001.

At the selected dose, AHR‐responsive carbon dot treatment significantly improved colon length, body weight, and DAI scores in DSS‐induced colitis mice (Figure 4A–D). Histological analysis showed better preserved epithelial integrity and crypt architecture, with reduced goblet cell loss and inflammatory cell infiltration after carbon dot treatment (Figure 4E,F). Moreover, the expression of inflammatory factors including IL‐17A and MPO were significantly decreased in carbon dot‐treated groups compared with colitis group (Figure 4G,H). Notably, the expression of AHR in the colon was markedly increased following carbon dot treatment (Figure 4I). The expression of FOXP3 in the colon was significantly increased after carbon dot treatment (Figure 4J). The proportion of Foxp3^+^ Treg cells in the spleen and lymph node was not significantly altered by the carbon dot administration (Figure S8A). Thus, we measured the expression of tissue resident Treg cells in colon by staining FOXP3 and ST2 [29]. The results revealed a significant increase in ST2^+^FOXP3^+^double‐positive cells after carbon dot treatment in both control and DSS‐induced colitis groups, indicating an increase in Treg cell populations, and most of them are tissue resident‐Treg (Figure 4J,K). To examine whether the therapeutic effects of carbon dots specifically target the colon and intestine, we tracked the intrinsic fluorescence of carbon dots in vivo. Biodistribution imaging revealed preferential accumulation of carbon dots in the gastrointestinal tract of colitis mice compared with control ones (Figure 4L,M). These findings suggest that carbon dots attenuate DSS‐induced colitis by driving gut resident Treg cells.

### 2.5 AHR‐Responsive Carbon Dots Accelerated Skin Wound Healing in Colitis Mice

**FIGURE 5:**
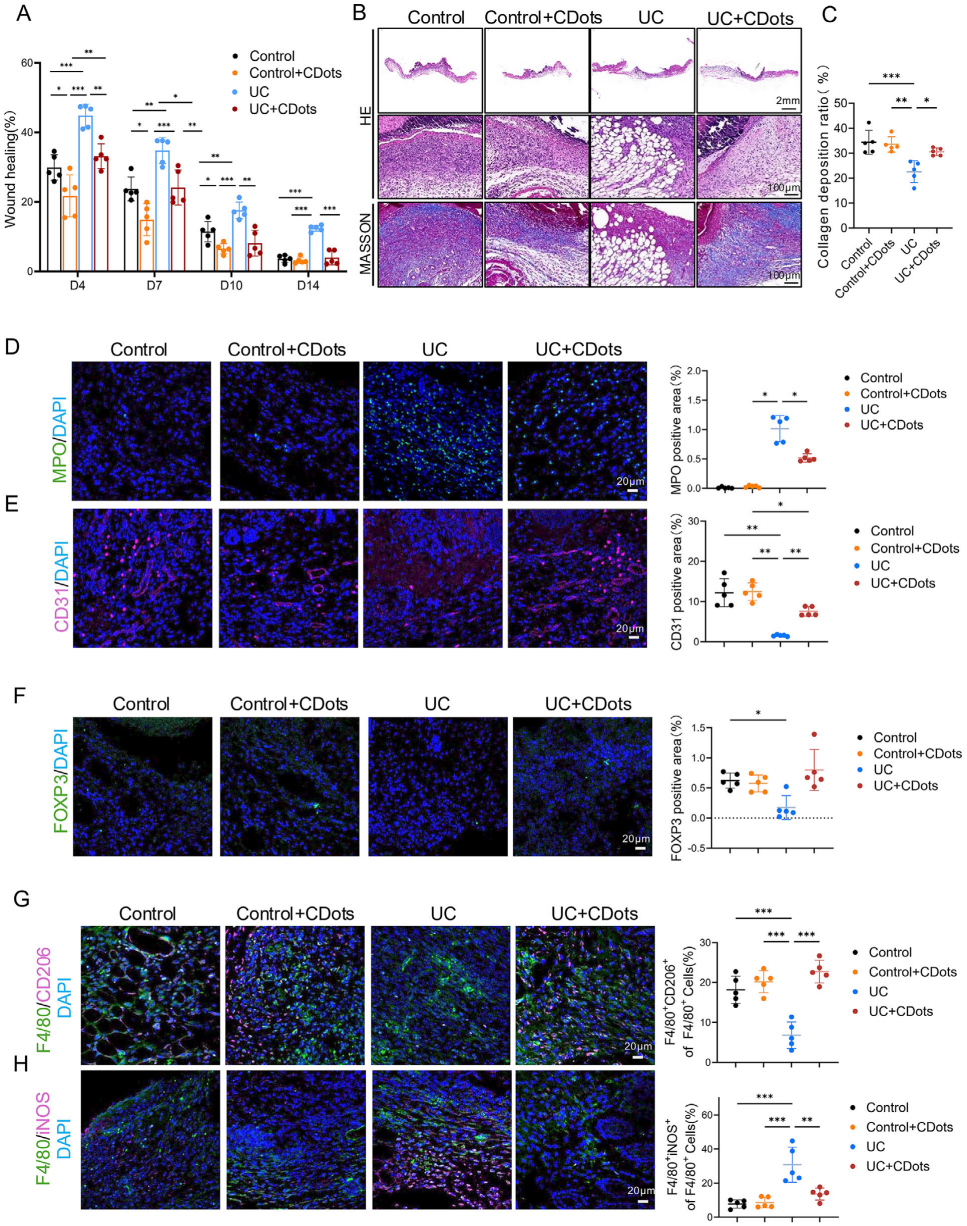
AHR‐responsive carbon dots accelerated skin wound healing in colitis mice. (A) Quantification of the skin wound healing rates at day 4, 7, 10, and 14 post‐injury in control and DSS‐induced colitis mice with or without carbon dot treatment. (B, C) The histological structure and collagen deposition of skin wound healing lesion after 7 days injury, as assessed by H&E and Masson's trichrome staining. (D‐F) The expression and quantification of MPO (D), CD31 (E) and FOXP3 (F) in the skin lesion after 7 days injury in control and DSS‐induced colitis mice with or without carbon dot treatment, as assessed by immunofluorescence staining. (G, H) The infiltration and quantification of CD206^+^ (G) and iNOS^+^ (H) macrophages in the skin lesion after 7 days injury in control and DSS‐induced colitis mice with or without carbon dot treatment, as assessed by immunofluorescence staining. F4/80^+^ cells (green) represented macrophages. Data are presented as mean ± SD. n = 5 mice per group. CDots, carbon dots. UC, DSS‐induced colitis. *p < 0.05, **p < 0.01, ***p < 0.001.

To investigate whether carbon dots regulates the extraintestinal manifestation of colitis, we established full‐thickness dorsal skin wounds in both control and DSS‐induced colitis mice. The results revealed markedly delayed skin wound healing in colitis mice compared with control ones. Whereas carbon dot treatment could significantly accelerate skin wound healing in colitis mice (Figures S9A and 5A). Remarkable wound healing with much smaller wound defects and well‐organized dermal structures was detected after carbon dot treatment (Figure 5B). Carbon dot treatment also increased collagen fiber density and thickness on the wound lesion edge (Figure 5B,C), indicating enhanced connective tissue reconstruction. Moreover, the expression of inflammatory factor MPO was decreased after carbon dot treatment (Figure 5D). Furthermore, enhanced neovascularization was detected after carbon dot treatment in skin lesion, as shown by higher expression of the vascular marker CD31 (Figure 5E). However, the carbon dot treatment failed to increase the expression of FOXP3 in the skin lesion (Figure 5F). We next analyzed the macrophages, which play a crucial role during tissue repair process. The results revealed that more pro‐inflammatory F4/80^+^ iNOS^+^ M1 macrophage subsets and fewer anti‐inflammatory F4/80^+^ CD206^+^ M2 macrophage subsets were infiltrated in the skin lesions of colitis mice compared with controls. Whereas carbon dot treatment increased M2 macrophages and decreased M1 macrophages, as assessed by immunofluorescence staining (Figure 5G,H).

Next, to determine whether carbon dots directly targeted local skin lesions, we tracked the distribution of carbon dots in vivo in colitis mice with skin wound. The results revealed that carbon dots predominantly accumulated in the gastrointestinal tract, while minimal signal was detected at the skin wound site (Figure S9B). These data revealed that carbon dots could accelerate skin wound healing in colitis mice, which may be regulated by the intestinal immune alteration that establish a pro‐regenerative niche.

### 2.6 Treg Cell‐Derived CCL1 Enhanced M2 Macrophage Polarization

**FIGURE 6:**
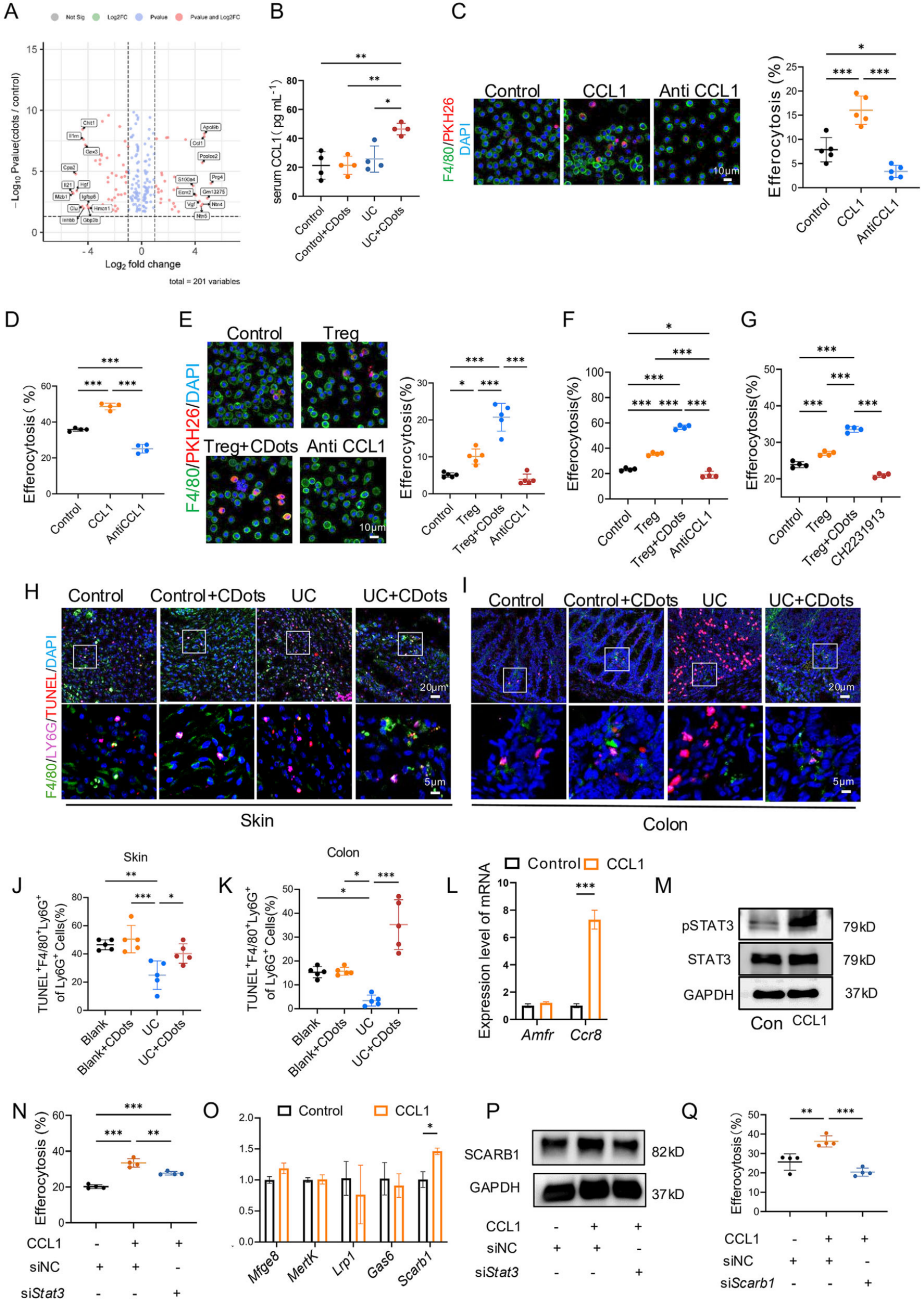
Treg‐derived CCL1 enhanced macrophage efferocytosis via STAT3‐SCARB1 signaling. (A) Volcano plot of differentially expressed cytokines in control and CDots‐treated T cells (fold change > 2, p < 0.05). (B) Serum CCL1 levels in control and DSS‐induced colitis mice with or without carbon dot treatment, as assessed by ELISA. (C, D) Efferocytosis by BMDMs in control, CCL1 or CCL1 neutralizing antibody treated groups, as assessed by immunofluorescence (C), and flow cytometry (D). (E, F) Efferocytosis by BMDMs in control, Treg cells and carbon dot‐treated‐Treg cells co‐culture groups with or without CCL1 neutralization, assessed by immunofluorescence (E), and flow cytometry (F). (G) Quantification of efferocytosis by BMDMs in control, Treg cells and carbon dot‐treated Treg cells coculture groups with or without AHR antagonist CH223191 administration, as assessed by flow cytometry. (H‐K) Representative immunofluorescence images and quantification of the ratio of TUNEL^+^ Ly6G^+^ F4/80^+^ positive cells in Ly6G^+^ cells, in the skin lesions (H, J) and colon tissues (I, K) from control and DSS‐induced colitis mice with or without carbon dot treatment. TUNEL^+^ cells (red) indicate apoptotic cells; F4/80^+^ cells (green) represented macrophages; and Ly6G^+^ cells (pink) represented neutrophils. (L) The gene expression of *Amfr* and *Ccr8* in control and CCL1 treated BMDMs, analyzed by RT‐qPCR. (M) The expression of p‐STAT3, total STAT3 protein and GAPDH in control and CCL1 treated BMDMs, as assessed by western blot. (N) Quantification of efferocytosis by BMDMs in control and *Stat3* siRNA treated groups, with or without CCL1 treatment, analyzed by flow cytometry. (O) The gene expression of *Mfge8, Mertk, Lrp1, Gas6* and *Scarb1* in control and CCL1 treated BMDMs, analyzed by RT‐qPCR. (P) The expression of SCARB1 of BMDMs in control and *Stat3* siRNA treated groups, with or without CCL1 administration, as analyzed by western blot. (Q) Quantification of efferocytosis by BMDMs in control and *Scarb1* siRNA treated groups, with or without CCL1 administration, analyzed by flow cytometry. Data are presented as mean ± SD. BMDMs, Bone marrow‐derived macrophages. CDots, carbon dots. UC, DSS‐induced colitis. *p < 0.05, **p < 0.01, ***p < 0.001.

Next, we explored how AHR‐responsive carbon dots regulated macrophage polarization to establish pro‐regenerative niche. First, we found that carbon dot treatment failed to alter macrophage M2 differentiation in vitro (Figure S10A,B). Thus, we further analyzed the RNA‐seq results comparing the gene expression between control and carbon dot‐treated groups. The results revealed that *Ccl1* was one of the top five increased genes after carbon dot treatment (Figure 6A). Moreover, *Ccl1* mRNA exprssion and CCL1 protein levels were significantly increased after carbon dot treatment, analyzed by RT‐qPCR and ELISA, respectively (Figure S10C,D). Furthermore, serum CCL1 levels were increased after carbon dot treatment (Figure 6B). CCL1 expression was elevated in colon tissues and skin lesions following carbon dot treatment (Figure S10E,F). Notably, the proportion of FOXP3^+^CCL1^+^ cells among total CCL1^+^ cells was increased in colon tissues, whereas no significant difference was observed in skin lesions (Figure S10G).

We next examined whether colon‐derived CCL1 regulated distal skin wound healing. A Cy5.5‐labeled anti‐CCL1 antibody was used as a fluorescent probe, and Cy5.5‐labeled rabbit IgG served as the isotype control [30]. Upon intramural injection of the antibody probe, no appreciable Cy5.5 signal was observed in the colonic tissue in the IgG‐treated control group. In mice administrated with anti‐CCL1 probe, colonic Cy5.5 fluorescence was detectable as early as at 1 h post‐injection and persisted up to 3 and 6 h. This colonic fluorescent signal was enhanced upon carbon dot treatment (Figure S11A,B). At skin wound sites, Cy5.5 fluorescence was undetectable at 1 h after anti‐CCL1 probe injection. Nevertheless, robust Cy5.5 signal emerged at 3 h, and this signal was augmented after carbon dot treatment (Figure S11C,D). Consistently, intravital live‐imaging demonstrated that Cy5.5‐tagged CCL1 infiltrated into skin wounds at 3 h, but not at 1 h, post‐probe injection (Figure S11E). These data indicate that CCL1 first accumulates within the colon and subsequently translocates to distant skin wounds, and carbon dot treatment facilitates the transport of CCL1.

Functionally, CCL1 treatment significantly induced M2 macrophage polarization, which was blocked by CCL1 antibody neutralization (Figure S12A). Moreover, Treg cells, particularly carbon dot treated‐Treg cells induced robust M2 macrophage polarization in a co‐culture system. This M2 macrophage polarization induced by Treg cells was also blocked by CCL1 neutralizing antibody treatment (Figure S12B). Additionally, these M2 macrophage polarization was partially abolished by AHR antagonist CH223191 treatment (Figure S12C). These results indicated that AHR‐responsive carbon dots promoted M2 macrophage polarization via Treg cell‐derived CCL1.

### 2.7 CCL1 Promoted Macrophage Efferocytosis via STAT3–SCARB1 Signaling

Accumulating evidence revealed that efferocytosis induces M2 polarization of macrophages [31]. To evaluate the effect of CCL1 on macrophage efferocytosis, PKH26‐labeled apoptotic neutrophils were co‐cultured with bone marrow‐derived macrophages (BMDMs) (Figure S13A). CCL1 increased the proportion of PKH26^+^F4/80^+^ macrophages, whereas CCL1 neutralization attenuated this response, as assessed by immunofluorescence staining and flow cytometry (Figure 6C,D). Treg cells also increased macrophage efferocytosis, and this effect was further enhanced when Treg cells had been treated with carbon dots. In contrast, CCL1 neutralization or AHR inhibition with CH223191 reduced Treg cell‐associated efferocytosis (Figure 6E–G). In skin lesions and colon tissues, carbon dot treatment increased the proportion of Ly6G^+^TUNEL^+^F4/80^+^ cells among total Ly6G^+^ cells (Figure 6H–K), consistent with enhanced clearance of apoptotic neutrophils in vivo. These results suggest that Treg cell‐derived CCL1 induces macrophage efferocytosis and reparative M2 macrophage responses.

Mechanistically, CCL1 stimulation significantly increased the expression of its receptor *Ccr8*, but not *Amfr* (Figure 6L). Furthermore, as STAT3 signaling has been identified as a downstream target of CCL1 [32], we confirmed that CCL1 treatment enhanced STAT3 phosphorylation in macrophages (Figures 6M and S13B). The macrophage efferocytosis induced by CCL1 treatment was inhibited by *Stat3* knockdown (Figures 6N and S13C), which indicated that CCL1 promotes macrophage efferocytosis via STAT3 signaling. Furthermore, the efferocytosis‐associated gene *Scarb1* was significantly increased after CCL1 treatment (Figure 6O). The increased expression of SCARB1 induced by CCL1 was attenuated by *Stat3* knockdown (Figures 6P and S13D). Functionally, the macrophage efferocytosis induced by CCL1 was reduced by *Scarb1* knockdown (Figures 6Q and S13E). Notably, the infiltration of SCARB1^+^ macrophages in the skin lesion was significantly increased after carbon dot treatment (Figure S13F). Collectively, these findings demonstrated that carbon dot treatment enhanced efferocytosis by CCL1‐STAT3‐SCARB1 mediated crosstalk between Treg cells and macrophages.

### 2.8 CCL1 Contributed to AHR‐Responsive Carbon Dot‐Associated Skin and Colonic Mucosal Repair

**FIGURE 7:**
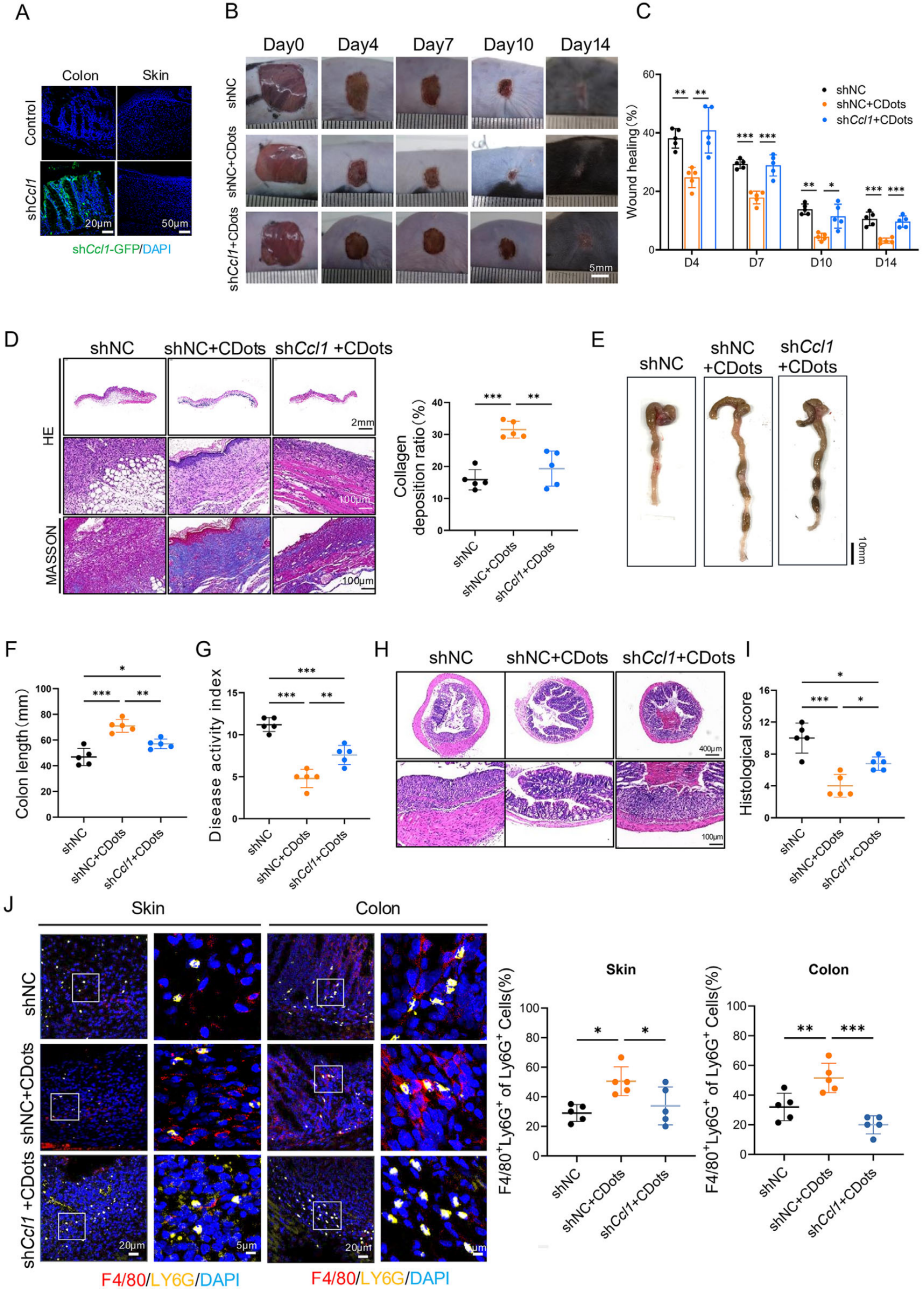
CCL1 contributes to carbon dot‐induced skin wound healing and colonic mucosal repair. (A) Representative fluorescence images of sh*Ccl1*‐GFP expression in colon and skin wound tissues after intramural colonic injection of the sh*Ccl1*‐GFP lentivirus. (B) Representative images of skin lesions at days 0, 4, 7, 10, and 14 post‐injury in DSS‐induced colitis mice treated by carbon dots with or without sh*Ccl1* lentivirus injection. (C) Quantification of the skin wound healing rate at day 4, 7, 10, and 14 post‐injury in DSS‐induced colitis mice treated by carbon dots with or without sh*Ccl1* lentivirus injection. (D) The histological structure and collagen deposition of skin wound healing lesion after 7 days injury, as assessed by H&E and Masson's trichrome staining (E) The representative images of colon in DSS‐induced colitis mice treated by carbon dots with or without sh*Ccl1* lentivirus injection. (F) The quantification of colon length in DSS‐induced colitis mice treated by carbon dots with or without sh*Ccl1* lentivirus injection. (G) The disease activity index scores in DSS‐induced colitis mice treated by carbon dots with or without sh*Ccl1* lentivirus injection. (H) Representative images of colons in DSS‐induced colitis mice treated by carbon dots with or without sh*Ccl1* lentivirus injection, as assessed by H&E staining. (I) The histological scores of DSS‐induced colitis mice treated by carbon dots with or without sh*Ccl1* lentivirus injection. (J) The efferocytosis, shown as F4/80^+^Ly6G^+^ double‐positive cells among Ly6G^+^ cells, in the skin lesion and colon tissues from DSS‐induced colitis mice treated by carbon dots with or without sh*Ccl1* lentivirus injection. F4/80^+^ cells (red) represent macrophages; and Ly6G^+^ cells (yellow) represented neutrophils. Data are presented as mean ± SD. n = 5 mice per group. CDots, carbon dots. UC, DSS‐induced colitis. sh*Ccl1, Ccl1*‐targeting shRNA lentivirus. shNC, non‐targeting control shRNA lentivirus. *p < 0.05, **p < 0.01, ***p < 0.001.

To assess whether CCL1 contributes to carbon dot‐mediated tissue repair, we knockdown CCL1 in colon tissues using lentiviral delivery of shRNA targeting the *Ccl1* gene. Three days after injection, GFP fluorescence was detected mainly in the colon but not the skin wound (Figure 7A). Compared with the carbon dot‐treated colitis group, *Ccl1* knockdown attenuated the pro‐healing effect of carbon dots, leading to delayed wound closure (Figure 7B,C) and reduced collagen deposition (Figure 7D). Moreover, the therapeutic effect of carbon dots on colitis was partially reduced by *Ccl1* knockdown, as shown by colon shortening and higher DAI scores compared with the carbon dot‐treated group (Figure 7E–G). Histological analysis showed increased edema, inflammatory cell infiltration, and basement membrane damage after *Ccl1* knockdown compared with the carbon dot‐treated group (Figure 7H,I). The carbon dot‐induced macrophage efferocytosis in colon and skin wound tissues was also reduced after *Ccl1* knockdown (Figure 7J). These results indicate that CCL1 is a critical mediator for Treg‐macrophage crosstalk to coordinate gut–skin immune repair.

Taken together, these findings indicate that AHR‐responsive carbon dots promote skin wound healing and colonic mucosal repair. Mechanistically, CCL1 induced by carbon dot treatment enhanced the crosstalk between Treg and macrophages, leading to macrophage efferocytosis mediated tissue regeneration.

## 3 Discussion

AHR is an essential regulator of the intestinal innate immune system, mediating microbial homeostasis and maintaining mucosal integrity. Endogenous low‐affinity AHR agonists are derived from a variety of dietary metabolites through microbial‐ or host‐mediated metabolism [33, 34]. However, the effectiveness of these metabolites and related first‐generation derivatives is limited by low potency, poor pharmacokinetic properties, and off‐target effects [35, 36]. To overcome these constraints, we employed pharmacophore modeling and fragment‐based drug design as a pre‐screening strategy to identify functional groups that contribute to AHR binding. Using the AHR ligand‐binding pocket, we developed a target‐specific training set and constructed pharmacophore models. Fragment‐based drug design analysis indicated that nitrogen‐ and oxygen‐containing aromatic rings enhance stable AHR binding. Based on the ability of carbon dots to incorporate diverse functional groups through precursor selection and synthesis conditions, we designed and synthesized AHR‐responsive carbon dots [37, 38, 39, 40]. These carbon dots featured a highly conjugated sp^2^ domain and heteroatom‐containing rings. Characterization by RNA‐seq, molecular dynamics simulations, and SPR demonstrated that these carbon dots bind AHR with an equilibrium dissociation constant (KD) of 2.35 µM and activate AHR signaling. Functionally, these AHR‐responsive carbon dots alleviated colitis, promoted gut mucosal repair, and accelerated skin wound healing in colitic mice.

Previous studies have shown that histidine‐derived carbon dots and glucose/D‐histidine‐derived carbon dots can alleviate experimental colitis in association with gut microbiota remodeling [41, 42]. In addition, manganese‐doped carbon dots were recently reported to alleviate sepsis‐associated lung injury by increasing microbiota‐derived indole‐3‐propionic acid, an endogenous AHR ligand [43]. Moreover, indole‐3‐aldehyde activates AHR‐dependent IL‐22‐mediated mucosal protection, whereas impaired microbial tryptophan metabolism into AHR ligands increases colitis susceptibility [44, 45]. Thus, whether carbon dots might indirectly activate AHR by modulating the gut microbiome requires further investigation.

We further identified a previously unrecognized ability of carbon dots to promote Treg cell differentiation from naïve CD4^+^ T cells. AHR‐responsive carbon dots significantly enhanced TGF‐β‐induced Treg polarization and were able to induce Treg differentiation even in the absence of exogenous TGF‐β. Previous studies have shown that the murine *Foxp3* promoter contains conserved and non‐conserved AHR‐binding sites, through which activated AHR can transactivate *Foxp3* transcription [15]. TGF‐β, a pivotal cytokine for Treg differentiation, exhibits a synergistic relationship with AHR both in vivo and in vitro. However, the exact mechanism underlying the Treg cell differentiation induced by AHR‐responsive carbon dots needs further investigation.

AHR agonists, including classic ligands such as FICZ or 2‐(1′H‐indol‐3′‐carbonyl)‐thiazole‐4‐carboxylic acid methyl ester (ITE), dietary agonists such as baicalein, and synthetic derivatives such as PY109 or ITE‐CONHCH_3_ have demonstrated efficacy in reducing disease severity in murine colitis models [27, 46, 47, 48]. However, many dietary AHR ligands show limited bioavailability because of rapid intestinal metabolism, whereas some synthetic AHR ligands have been developed to improve pharmacokinetic stability [49]. Nevertheless, the AHR‐activating effect of ITE may be transient, potentially due to the chemical instability of its methyl ester moiety in aqueous environments containing nonspecific esterases [46]. Low doses of FICZ and PY109 can also promote the production of the inflammatory cytokine IL‐17A, which may exert pro‐inflammatory effects in different contexts [35, 50]. Compared with small‐molecule AHR ligands, AHR‐responsive carbon dots may provide improved physicochemical stability and preferential colon retention. Functionally, AHR‐responsive carbon dots effectively induced gut‐resident ST2^+^Treg cells to attenuate colitis and accelerate skin wound healing.

The preferential accumulation of AHR‐responsive carbon dots in the inflamed colon may be associated with both inflammation‐associated tissue permeability and the physicochemical properties of the nanoparticles. In a DSS‐induced colitis model, nanoparticles have been shown to accumulate more efficiently in inflamed colonic lesions than in healthy tissues, supporting passive inflammation‐associated accumulation of nano‐systems in colitis [51]. This accumulation is generally attributed to the disrupted epithelial and vascular barriers in inflamed intestinal tissues, which increase nanoparticle access to the lesion site. After tissue entry, particle surface charge may further influence mucosal retention. The AHR‐responsive carbon dots developed in this study exhibited negative zeta potential of −9.529 mV. Previous studies using charged liposomes demonstrated that anionic nano‐systems adhered more strongly to inflamed colonic mucosa than neutral or cationic liposomes, suggesting that electrostatic interactions with altered inflammatory mucosal components may facilitate the adhesion of negatively charged nano‐systems to inflamed colon tissue [52, 53].

Consistent with their preferential intestinal accumulation and limited local accumulation at skin wound sites, AHR‐responsive carbon dot treatment did not markedly increase local Treg cell infiltration in skin wounds. However, it significantly enhanced macrophage efferocytosis and M2 macrophage polarization in the wound microenvironment. Mechanistically, we found that CCL1 is important for M2 macrophage polarization. Our results revealed that carbon dot stimulation enhanced CCL1 production by gut Treg cells, accompanied by elevated serum CCL1 concentrations. Moreover, higher CCL1 expression was detected in the skin wound lesion, which was not co‐localized with Treg cells in skin. These results indicate that CCL1 from gut Treg cells may mediate the crosstalk between gut and skin. It has been reported that fibronectin released into the circulation by activated peritoneal macrophages accelerated distal skin wound healing through an endocrine mechanism [54]. Besides, serum cytokines such as IL‐10 and IL‐22 have been linked to gut barrier dysfunction and skin inflammation in atopic dermatitis [55]. Although colon‐derived CCL1 take participate the distal skin wound healing, how gut Treg‐derived CCL1 acts through the circulation and modulates skin immune niche requires further investigation.

Furthermore, we found that CCL1 activated the STAT3–SCARB1 signaling pathway in macrophages, thereby enhancing their efferocytic capacity. In contrast, CCL1 treatment did not markedly alter the expression of several other efferocytosis‐related molecules, including MerTK, Gas6, MFGE8, and LRP1. These findings suggest that the CCL1–STAT3–SCARB1 axis represents a less‐recognized mechanism by which intestinal immune signals regulate macrophage‐mediated tissue repair at distal skin sites. Although efferocytosis has been reported to activate AHR signaling in macrophages and promote atherosclerosis regression [56], whether AHR activation induced by AHR‐responsive carbon dots further participates in a positive feedback loop during efferocytosis remains to be explored.

## 4 Conclusions

In summary, we developed functionalized carbon dots with AHR‐responsive properties to modulate the Treg‐macrophage crosstalk. Functionally, AHR‐responsive carbon dots achieved cross‐organ anti‐inflammatory and pro‐reparative effects, orchestrating skin and colonic mucosal wound healing. This work not only provides a novel strategy for treating IBD and its extraintestinal manifestations, but also establishes a design strategy for AHR‐mediated immune reprogramming, advancing the development of therapeutic nanomaterials (Figure S14).

## 5 Materials and Methods

### 5.1 Animals

C57BL/6J mice (6–8 weeks of age) were obtained from the Department of Laboratory Animal Science, Peking University Health Science Center. All animal procedures were approved by the Institutional Animal Care and Use Committee of Peking University (approval no. LA2022476). Mice were maintained under specific pathogen‐free (SPF) conditions at 25°C with 60% humidity in a 12‐h light/dark cycle.

### 5.2 Antibodies and Cytokines

Details of the Antibodies and Cytokines Used in this Study Were Listed in Table S1.

### 5.3 Single‐Cell RNA Sequencing Analysis

Four publicly available single‐cell RNA sequencing datasets (GSE95459, GSE114374, GSE116222, GSE182270) [22, 23, 24] related to IBD were integrated and analyzed to systematically construct a single‐cell atlas of the IBD microenvironment. Raw expression matrices were converted into Seurat objects with harmonized metadata. Rigorous quality control was performed, including per‐sample doublet removal using DoubletFinder and filtering of low‐quality cells (500–6000 detected genes and mitochondrial gene proportion <15%). Batch effects across samples were corrected with Harmony, and principal component analysis (PCA)‐based integration was used for downstream analyses. Using canonical marker genes, we identified 9 major cell types: T cells, B cells, macrophages, plasma cells, stromal cells, pericytes, glial cells, colonocytes, and endothelial cells. The T‐cell compartment was subsequently isolated and automatically annotated using the scType database [57]. Cell clusters were scored based on positive and negative marker gene sets, and low‐confidence annotations were filtered by the default score threshold. Based on ScType annotation and established marker gene profiles, we further annotated 9 distinct T/NK‐cell populations, including naïve T cells, CD4^+^ naïve T cells, CD4^+^ central memory T cells, regulatory T cells, CD4^+^ effector memory T cells, CD8^+^ effector memory T cells, CD8^+^ tissue‐resident memory T cells, CD8^+^ natural killer T‐like cells, and natural killer cells. Sample S90 contained no cells passing this selection criterion and was excluded from subsequent analyses. Differential expression analysis comparing IBD and control groups (|log2FC| > 1 and FDR‐adjusted *p* < 0.05), followed by KEGG pathway enrichment, systematically delineated the molecular signatures and functional alterations of T‐cell subsets in IBD.

### 5.4 RNA‐seq Analysis

Total RNA was extracted from control and carbon dot‐treated T cells and submitted to Beijing Genomics Institution for transcriptome sequencing. Three biologically independent samples were included in each group. RNA libraries were prepared from 1 µg of total RNA per sample using the NEBNext Ultra RNA Library Prep Kit, followed by paired‐end sequencing on an Illumina HiSeq 2500 platform with 2 × 100 bp reads.

Raw sequencing data were converted into FASTQ files using bcl2fastq software version 1.8.4. After adaptor removal and quality filtering, clean reads were mapped to the mouse reference genome GRCm38/mm10. Transcript assembly and expression quantification were performed using DNASTAR Lasergene version 15.0, and gene expression levels were calculated as FPKM values. Genes with a |log_2_ fold change| > 1 and adjusted p < 0.05 were defined as differentially expressed genes. KEGG pathway enrichment analysis was performed for differentially expressed genes, and gene set enrichment analysis was used to evaluate pathway‐level changes between control and carbon dot‐treated T cells. The RNA‐seq data have been deposited in the GSA database (No. CRA052803).

### 5.5 Generation of Ligand‐Based Common‐Feature Pharmacophore Models

To define structural features potentially involved in AHR recognition, a ligand‐based pharmacophore analysis was performed using a curated set of reported AHR ligands. The 2D chemical structures of six AHR antagonists and ten AHR agonists were retrieved from the MedChemExpress database and converted into 3D conformations using Avogadro. Energy minimization was then carried out with the CHARMM force field before conformational sampling. For each ligand, up to 255 conformers were generated within an energy window of 20 kcal mol^−^^1^. When stereoisomers were present, conformer generation was conducted independently for each isomer. Common‐feature pharmacophore hypotheses were generated using the HipHop algorithm. Ten candidate models were generated, and the two top‐ranked models with the best ligand–feature alignment were selected for subsequent analysis.

### 5.6 Fragment‐Based Drug Design

A fragment‐based screening workflow was used to identify chemical fragments potentially involved in AHR recognition. The ligand set consisting of six AHR antagonists and ten AHR agonists (Figure S2) was fragmented using the Retrosynthetic Combinatorial Analysis Procedure (RECAP). Fragmentation was performed using the standard RECAP disconnection rules for synthetically accessible bonds, including amide, ester, amine, urea, ether, and related linkages. The AlphaFold‐predicted AHR structure was obtained from the UniProt database (ID: P35869), and the putative binding pocket was defined before fragment screening. The Ludi algorithm was then applied to evaluate the spatial fit and chemical complementarity between the generated fragments and the AHR binding site. Candidate fragments predicted to interact favorably with AHR were retained, and their spatial distribution within the binding pocket was visualized using ChimeraX. This workflow was adapted from a previously reported computational screening strategy, with modifications for AHR‐targeted carbon dot design [58].

### 5.7 Synthesis of Carbon Dots

Carbon dots were synthesized by microwave‐assisted carbonization using ascorbic acid and polyethyleneimine as precursors. Briefly, ascorbic acid (0.5 g, Sigma‐Aldrich) and polyethyleneimine (0.125 g, PEI, MW  =  25 kDa, Sigma‐Aldrich) were dissolved in 10 mL PBS and ultrasonicated for 3 min to form a homogeneous solution. The solution was then heated in a microwave oven at 700 W for 5 min. After cooling to room temperature, the reaction product was diluted with 40 mL deionized water and centrifuged at 5200 × g for 5 min to remove large aggregates. The supernatant was passed through a 0.22 µm membrane and dialyzed against deionized water using a dialysis membrane with a molecular weight cut‐off of 3.5 kDa for 7 days. The purified carbon dot solution was lyophilized to obtain carbon dots in powder form.

### 5.8 Characterization of Carbon Dots

The morphology of the carbon dots was examined by transmission electron microscopy (TEM; Hitachi H‐800, 200 kV, Hitachi, Japan) and high‐resolution TEM (HRTEM; JEM‐2100F, 200 kV, JEOL, Japan). The zeta potential was measured using a Malvern Panalytical zeta potential analyzer equipped with a DTS1070 cell at 25°C, with water used as the dispersant. UV‐vis absorption spectra were recorded on a Lambda 800 spectrophotometer, and fluorescence spectra were obtained using a Shimadzu RF‐5301 PC spectrophotometer (Shimadzu, Japan). Surface functional groups were analyzed by Fourier transform infrared spectroscopy using a Nicolet AVATAR 360 instrument (Analytical Instruments, USA). Elemental composition and chemical bonding states were determined by X‐ray photoelectron spectroscopy using a VG ESCALAB MKII spectrometer with Mg Kα radiation (1253.6 eV; Thermo Fisher Scientific, USA).

### 5.9 Cell Culture and T‐Cell Differentiation

Single‐cell suspensions were prepared from the spleens and peripheral lymph nodes of 8‐week‐old C57BL/6J mice. CD4^+^ T cells were first enriched using CD4 MicroBeads (Miltenyi Biotec). Then, naïve CD4^+^T cells, defined as CD4^+^CD25^−^CD62L^hi^CD44^lo^ cells, were purified by flow cytometric sorting (FACSAria III, BD Biosciences). Sorted cells were seeded in 48‐well plates at 0.5 × 10^6^ cells per well and stimulated with plate‐bound anti‐CD3 antibody (5 µg mL^−1^) and soluble anti‐CD28 antibody (5 µg mL^−1^) in complete RPMI 1640 medium containing 10% fetal bovine serum, 1% penicillin‐streptomycin, 1% L‐glutamine, 10 mM HEPES, 1% sodium pyruvate, and 50 µM β‐mercaptoethanol.

For T cell differentiation, cytokines were added at the beginning of culture. Th1 polarization was induced with mouse IL‐2 (25 U mL^−1^) and mouse IL‐12 (15 ng mL^−1^). Th17 polarization was induced with human TGF‐β (1 ng mL^−1^) and mouse IL‐6 (20 ng mL^−1^). Treg differentiation was induced with human TGF‐β (2 ng mL^−1^) and mouse IL‐2 (25 U mL^−1^). After four days of culture, cells were collected for flow cytometry, RT‐qPCR, western blotting, or other downstream analyses.

For intracellular cytokine staining, T cells were restimulated for 4 h at 37°C with PMA (50 ng mL^−1^), ionomycin (500 ng mL^−1^), and GolgiStop. Cells were then fixed and permeabilized using the Foxp3/Transcription Factor Staining Buffer Set (Thermo Fisher Scientific) according to the manufacturer's protocol. Flow cytometric data were acquired on a BD LSRFortessa and analyzed with FlowJo software version 10.8.1.

Bone marrow‐derived macrophages were generated from femurs and tibias of 8‐week‐old C57BL/6J mice. Bone marrow cells were flushed out and cultured in DMEM supplemented with 10% fetal bovine serum and 1% penicillin‐streptomycin. M‐CSF (50 ng mL^−^^1^) was added for 7 days to promote macrophage differentiation. For M2 macrophage polarization, cells were treated with IL‐4 at 20 ng mL^−1^ for 24 h.

### 5.10 Cell Viability Assay

CD4^+^ T cells were stimulated for 24 h, then seeded in 96‐well plates and treated with carbon dots at concentrations of 0, 100, 200, or 400 µg mL^−1^ for 24 h. The viability of CD4^+^ T cells was evaluated using a Cell Counting Kit‐8 (CCK‐8) assay.

### 5.11 Carbon Dots Internalization Assay

For immunofluorescence microscopy, activated CD4^+^ T cells were labeled with DiD (Invitrogen) according to the manufacturer's instructions. They were then incubated with carbon dots (200 µg mL^−1^) for 0.5 h, 2 h or 12 h at 37 °C. After incubation, cells were washed twice with PBS and imaged using an Olympus FV3000 inverted fluorescence microscope. For quantitative analysis, CD4^+^ T cells were incubated with carbon dots (200 µg mL^−1^) for 0.5, 2, 4, 6, 12, 24, 48, or 72 h. Cells were then washed twice with PBS and analyzed on a BD LSRFortessa flow cytometer (BD Biosciences).

### 5.12 Flow Cytometric Analysis of Apoptosis

Cell apoptosis was assessed using an Annexin V‐FITC/propidium iodide (PI) apoptosis detection kit (Beyotime Biotechnology, C1062M, China) according to the manufacturer's instructions. Briefly, harvested cells were washed twice with PBS and resuspended in 100 µL binding buffer. Cells were then incubated with 5 µl Annexin V‐FITC and 5 µL PI for 15 min at room temperature in the dark, followed by analysis on a flow cytometer (CytoFLEX, Beckman Coulter, USA).

### 5.13 Cell Proliferation Analysis by Flow Cytometry

Cell proliferation was assessed using a PE‐Ki67 kit (BD, Catalog # 557503) according to the manufacturer's instructions. Briefly, harvested cells were washed twice with PBS and resuspended in 100 µL binding buffer. The cells were then permeabilized and incubated with 5 µL of Ki67 antibody for 30 min at 4°C in the dark. Following incubation, cells were analyzed on a BD LSR Fortessa (BD Biosciences, USA)

### 5.14 Immunofluorescence Staining

Briefly, tissue sections were subjected to enzyme‐mediated antigen retrieval for 30 min at 37°C and blocked with 5% bovine serum albumin for 1 h at room temperature. Primary antibodies were diluted according to the manufacturers’ instructions and applied to the sections overnight at 4°C. After three washes with PBS, Alexa Fluor 488‐, Alexa Fluor 594‐, or Alexa Fluor 647‐conjugated secondary antibodies (Invitrogen) were added for 1 h at room temperature in the dark. Nuclei were counterstained with DAPI‐containing mounting medium. Images were captured using an Olympus FV3000 fluorescence microscope. Fluorescence signals were quantified with ImageJ using the same threshold settings for all images within the same staining experiment.

### 5.15 Real‐Time Polymerase Chain Reaction (RT‐qPCR)

Total RNA of T cells was extracted with Trizol reagent (Invitrogen, 15596026, USA), and cDNA was synthesized using the PrimeScript RT kit (Takara, RR037A, Japan). qPCR was performed with SYBR Green Supermix (Bio‐Rad, 1708880, USA). Gene‐specific primers were listed in Table S2. The level of mRNA expression for each gene was normalized to *β‐Actin*.

### 5.16 Western Blotting

Cells were lysed in RIPA buffer supplemented with 1% PMSF, and protein concentrations were determined before loading. For each sample, 30 µg of total protein was separated by SDS‐PAGE and transferred onto PVDF membranes (Millipore, Billerica, MA, USA). Membranes were blocked with 5% non‐fat milk or 5% BSA in TBST, depending on the primary antibody, for 1 h at room temperature. The membranes were incubated with primary antibodies at the manufacturers recommended dilutions overnight at 4°C, followed by HRP‐conjugated secondary antibodies (1:5000; ZSGB‐Bio, Beijing, China) for 1 h at room temperature. Bands were visualized using SuperSignal West Pico PLUS chemiluminescent substrate (Thermo Fisher Scientific, Waltham, MA, USA) and quantified using ImageJ software (Figures S15 and S16).

### 5.17 Molecular Dynamics Simulations

The AlphaFold‐predicted AHR structure was obtained from the UniProt database (ID: P35869), and its stereochemical quality was evaluated using Ramachandran plot analysis. Based on HRTEM and XPS characterization, a simplified structural model of the carbon dots was constructed and optimized by density functional theory calculations. Molecular dynamics simulations of the AHR–carbon dot complex were performed using GROMACS version 2018.8 with the AMBER99SB‐ILDN force field. The complex was placed in a periodic cubic box, solvated with TIP3P water molecules, and neutralized with Cl^−^ ions. After energy minimization, the system was equilibrated under NVT and NPT ensembles at 300 K and 1.0 bar, respectively. A 120‐ns production simulation was then performed without positional restraints. Trajectories were saved every 10 ps and analyzed for root mean square deviation (RMSD) and root mean square fluctuation (RMSF).

### 5.18 Surface Plasmon Resonance Assay (SPR)

SPR assays were conducted to evaluate the interaction between purified recombinant AHR proteins and carbon dots using a Biacore 8K instrument (Cytiva). The His‐tagged human AHR protein was expressed in *Escherichia coli* (CSB‐EP001481HU3, CUSABIO). Briefly, AHR was immobilized via amine coupling after EDC/NHS activation, followed by ethanolamine blocking. Serial dilutions of carbon dots (5–100 µM in PBS) were injected at 30 µL/min (120 s association/180 s dissociation). The surface was regenerated with 10 mM glycine‐HCl (pH 2.0). Data were reference‐subtracted and fitted to a 1:1 binding model to obtain kinetic parameters. All measurements were performed in triplicate at 25°C. The equilibrium dissociation constant (KD) was calculated using the Biacore 8 K Evaluation Software, employing appropriate fitting models to ensure accuracy.

### 5.19 In Vitro Efferocytosis Assay

Neutrophils were isolated from the femurs of 8‐week‐old mice using a commercial neutrophil isolation kit (Solarbio, China) according to the manufacturer's instructions. Briefly, neutrophils were labeled with PKH26 (Sigma Aldrich, USA) and co‐incubated with BMDMs for 1 h at 37°C. After incubation, cells were fixed with 4% paraformaldehyde and stained with anti‐mouse F4/80. Efferocytosis was quantified by flow cytometry using a BD LSRFortessa (BD Biosciences, USA) or imaged by fluorescence microscopy using an Olympus FV3000.

### 5.20 SiRNA Transfection

BMDMs were seeded at 2 × 10^5^ cells per well in six‐well plates and transfected with 50 nM of vehicle, Stat3, or Scarb1 siRNAs using the riboFECT CP Transfection Kit (Ribobio, C10511 05) as per the manufacturer's protocol. Knockdown efficiency was assessed at 48 h post‐transfection using RT‐qPCR and Western blotting. Gene‐specific primers were listed in Table S2.

### 5.21 In Vivo Biodistribution of Carbon Dots

8‐week‐old C57BL/6 J mice were intravenously injected with carbon dots to evaluate in vivo biodistribution. The samples were harvested at 3, 6, 12, and 24 h injections. PBS‐injected mice were used as controls. Fluorescence signals were detected using an In Vivo Imaging System (IVIS) Spectrum imaging system (PerkinElmer, USA) and quantified with Living Image software (PerkinElmer, USA; version 4.3.1).

### 5.22 Dextran Sodium Sulfate (DSS)‐Induced Ulcerative Colitis Model

Acute colitis was induced in 8–10‐week‐old C57BL/6 J mice by administration of 2% (w/v) DSS (MP Biomedicals, USA; Cat. no. 160110) in drinking water for 14 days. Control animals received water alone. From the first day of DSS treatment, carbon dots were administered via tail vein injection at 10, 20 and 40 mg kg^−1^ per injection on days 1, 4, 7, and 10 for a total of four injections. Body weight was recorded daily. Samples were harvested after 14 days of DSS administration for further analysis. The disease activity index (DAI) was calculated based on weight loss (0, 1, 2, 3 or 4 points for <1%, 1–5%, 5–10%, 10–15%, or >15% loss, respectively), stool consistency (0, 2 or 4 points for normal, loose or watery stools, respectively) and the presence of bleeding (0, 2 or 4 points for none, mild or gross bleeding, respectively). The sum of these scores was used as the DAI score.

### 5.23 In Vivo Biosafety Evaluation of Carbon Dots

Healthy C57BL/6J mice were intravenously administered PBS or carbon dots at doses of 10, 20, or 40 mg kg^−1^ on days 1, 4, 7, and 10. On day 14, the blood was obtained for blood routine and biochemical analysis, and the major organs were collected for HE staining

### 5.24 Wound Healing Mouse Model

The skin wound healing model was established in the ulcerative colitis mice. Briefly, 2% DSS was administered in drinking water for 14 days to induce colitis‐associated delayed wound healing. A full‐thickness dorsal skin wound (1 cm × 1 cm) was created after 2 days of DSS administration. Wound closure was monitored for 14 days after wound creation. For the carbon dot‐treated group, carbon dots were administered via tail vein injection at 20 mg kg^−^^1^ per injection on days 1, 4, 7, and 10 after the start of DSS treatment for a total of four injections over the 14‐day experimental period. For histological and immunofluorescence analyses of skin wound tissues, wound samples were collected on day 7 after wound creation. For CCL1 knockdown experiments, pClinti‐U6‐shRNA (NC)‐CMV‐EGFP‐WPRE (OBiO, GL404NC, China) or pClinti‐U6‐shRNA (CCL1)‐CMV‐EGFP‐WPRE (OBiO, Y24188, China) was injected intramurally into the colon (20 µL, 1 × 10^7^ TU/mL). Briefly, mice were anesthetized and placed under sterile conditions. Following a midline incision, the colon was exposed and the lentiviral suspension was injected into the colonic wall using a 30‐gauge needle. The incision was closed with sutures [59]. The sequence for CCL1 shRNA was: CCGGGGTATTCAGGCTGAACAAAGGCTCGAGCCTTTGTTCAGCCTGAATACCTTTTTTG.

Following the 3R principle of animal experimentation (Reduction, Replacement, and Refinement) [60], the sample size was estimated using the resource equation method. The error degree of freedom (E) value between 10 and 20 is generally considered acceptable for animal studies [61]. In our therapeutic experiment, four groups with five mice per group were used, resulting in an E value of 16, which falls within the recommended range. Therefore, five mice per group were considered appropriate for detecting the main therapeutic effects while minimizing unnecessary animal use.

### 5.25 CCL1‐Targeted Fluorescence Tracing

Anti‐mouse CCL1 rabbit recombinant antibody (Proteintech, 98502‐2‐RR) and control rabbit IgG were separately labeled with Cy5.5 using a Cyanine5.5 Labeling Kit (Elabscience, E‐LK‐C004A) according to the manufacturer's instructions. DSS‐induced colitis mice with or without carbon dot treatment received intramural injections of Cy5.5‐labeled anti‐CCL1 antibody or Cy5.5‐labeled rabbit IgG into the colonic wall at a dose of 20 µg in 20 µL per mouse using a 30‐gauge needle [30, 59]. In vivo fluorescence images were acquired at 1, 3 and 6 h after injection using an IVIS imaging system. At 1, 3, 6 h, mice were sacrificed, and colon tissues and skin wound tissues were collected for immunofluorescence analysis.

### 5.26 Hematoxylin and Eosin (H&E) Staining and Masson's Trichrome Staining

The samples were fixed in 4% paraformaldehyde at 4°C overnight, dehydrated through a graded ethanol series, embedded in paraffin, and sectioned at 5 µm thickness. Sections were deparaffinized in xylene, rehydrated through descending ethanol concentrations, and stained with hematoxylin for 5 min, followed by eosin for 1–2 min. After dehydration and clearing, slides were mounted with neutral resin and examined under a light microscope (Leica Microsystems). The histological score for DSS‐induced colitis was assessed using the scoring system shown in Table S3.

For Masson's Trichrome staining, paraffin‐embedded tissue sections (5 µm) were deparaffinized, rehydrated, and processed using a standard Masson's Trichrome Staining Kit (Solarbio, China) according to the manufacturer's protocol. The sections were stained with Weigert's iron hematoxylin, followed by counterstaining with aniline blue. Images were acquired using a light microscope (Leica Microsystems). To evaluate collagen deposition, the ratio of collagen‐positive areas was quantified using ImageJ.

### 5.27 ELISA Assay

To determine serum CCL1 level, mouse serum was collected. To measure CCL1 level secreted by Treg cells, cell culture supernatants were collected after centrifuging at 4000 × g for 20 min. Briefly, cytokine levels were measured using a double‐antibody sandwich ELISA kit for CCL1 (Solarbio, China; Cat. SEKM‐0134). Absorbance at 450 nm was read on a microplate reader (Molecular Devices, USA).

### 5.28 Statistical Analysis

Statistical analyses were performed using SPSS (version 22.0). Data are presented as mean ± SD. For comparisons between two groups, unpaired two‐tailed Student's t‐tests were used. For comparisons among more than two groups, one‐way ANOVA followed by Tukey's multiple‐comparison test was applied. P < 0.05 was considered statistically significant.

## Author Contributions

R.Y. contributed to the conception and design, data acquisition, analysis, and interpretation, and critically revised the manuscript. Z.Z. contributed to the design, data analysis, and interpretation, and critically revised the manuscript. H.Z. contributed to the analysis of single‐cell sequencing data and critically revised the manuscript. S.L. contributed to data analysis and critically revised the manuscript. X.X. and W.Z. contributed to nanoparticle synthesis and critically revised the manuscript. X.L. critically revised the manuscript. All authors gave final approval and agree to be accountable for all aspects of the work.

## Funding

This work was supported by the National Key Research and Development Program of China No. 2022YFA1105800 (R.Y.), The Beijing High‐Level Innovation and Entrepreneurship Talent Support Program Leading Talent Projects No. G202512016 (R.Y.), Beijing Municipal Natural Science Foundation (L232107), the Fundamental Research Funds for the Central Universities‐Peking University Clinical Scientist Training Program of BMU2024PYJH019.

## AI Use and Graphic Preparation Statement

No AI tools were used to generate experimental data, figures, data analysis, scientific conclusions, or interpretations of this manuscript. ChatGPT 5.5 was used only to assist with language and grammar checking. The Table of Contents graphic was created with BioRender. The chemical structures were created by ChemDraw 22.2.

## Conflicts of Interest

The authors declare no conflicts of interest.

## Supporting information




**Supporting File**: advs77526‐sup‐0001‐SuppMat.pdf.

## Data Availability

The data that support the findings of this study are available on request from the corresponding author. The data are not publicly available due to privacy or ethical restrictions.
