# Supplementary material for: AHR‐Responsive Carbon Dots Orchestrate Skin Wound Healing and Colonic Mucosal Repair via Treg‐Mediated Macrophage Efferocytosis
Source: Adv Sci (Weinh). 2026 Sep 6:e77526. Online ahead of print. doi: 10.1002/advs.77526 (PMC13547795; doi:10.1002/advs.77526)
Supplement: Supplementary file 1 [file ADVS-9999-e77526-s001.pdf]

Supplementary Materials for

AHR-responsive carbon dots orchestrate skin wound healing and colonic mucosal repair *via* Treg-mediated macrophage efferocytosis

Zilu Zhu[1], Han Zhang[1], Shuai Lin[1], Wenqian Zheng[2], Xin Li[3] , Xiaowei Xu[4*], Ruili Yang[1*]

[1]Department of Orthodontics, Peking University School and Hospital of Stomatology, National Center for Stomatology & National Clinical Research Center for Oral Diseases & National Engineering Research Center of Oral Biomaterials and Digital Medical Devices，Beijing Key Laboratory of Digital Stomatology & NHC Key Laboratory of Digital Stomatology & NMPA Key Laboratory for Dental Materials Beijing, 100081,China

[2]Jilin Provincial Key Laboratory of Tooth Development and Bone Remodeling, Hospital of Stomatology, Jilin University, Changchun 130021, China

[3]Division of Restorative Dental Sciences, Faculty of Dentistry, The University of Hong Kong, Hong Kong SAR, China.

[4]Department of Stomatology, The First Hospital of Jilin University, Jilin University, Changchun 130021, China

Corresponding Authors:

Ruili Yang, Department of Orthodontics, Peking University School and Hospital of Stomatology, 22 Zhongguancun Avenue South, Haidian District, Beijing, 100081, China. Email: ruiliyang@bjmu.edu.cn

Xiaowei Xu, Department of Stomatology, The First Hospital of Jilin University, Jilin University, Changchun 130021, China

Email：xiaoweixu@jlu.edu.cn

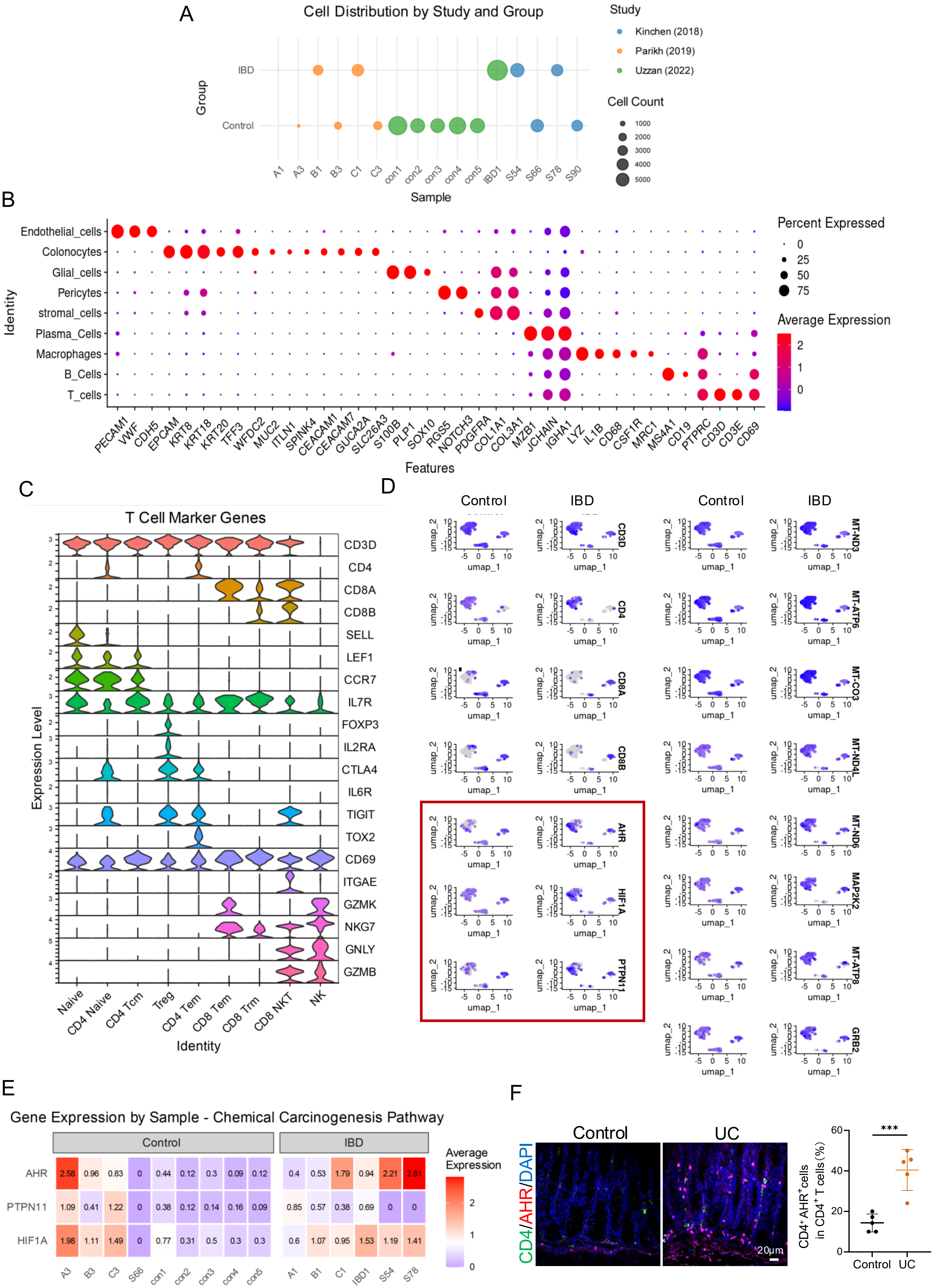
A
Cell Distribution by Study and Group
Study
Kinchen (2018)
Parikh (2019)
Uzzan (2022)
Cell Count
1000
2000
3000
4000
5000
Group
IBD
Control
A1 A3 B1 B3 C1 C3 con1 con2 con3 con4 con5 IBD1 S54 S66 S78 S90
Sample
B
Endothelial_cells
Colonocytes
Glial_cells
Pericytes
stromal_cells
Plasma_Cells
Macrophages
B_Cells
T_cells
Identity
PECAM1 VWF CDH5 EPCAM KRT8 KRT18 KRT20 TFF3 WFDC2 MUC2 ITLN1 SPINK4 CEACAM1 CEACAM7 GUCA2A SLC26A3 S100B PLP1 SOX10 RGS5 NOTCH3 PDGFRA COL1A1 COL3A1 MZB1 JCHAIN IGHA1 LYZ IL1B CD68 CSF1R MRC1 MS4A1 CD19 PTPRC CD3D CD3E CD69
Features
Percent Expressed
0
25
50
75
Average Expression
2
1
0
C
T Cell Marker Genes
CD3D
CD4
CD8A
CD8B
SELL
LEF1
CCR7
IL7R
FOXP3
IL2RA
CTLA4
IL6R
TIGIT
TOX2
CD69
ITGAE
GZMK
NKG7
GNLY
GZMB
Expression Level
Naive CD4 Naive CD4 Tcm Treg CD4 Tem CD8 Tem CD8 Trm CD8 NKT NK
Identity
D
Control
IBD
Control
IBD
CD3D
CD4
CD8A
CD8B
AHR
HIF1A
PTPN11
MT-ND3
MT-ATP6
MT-CO3
MT-ND4L
MT-ND6
MAP2K2
MT-ATP8
GRB2
umap_1
umap_2
E
Gene Expression by Sample - Chemical Carcinogenesis Pathway
Control
IBD
AHR 2.58 0.96 0.83 0 0.44 0.12 0.3 0.09 0.12 0.4 0.53 1.79 0.94 2.21 2.81
PTPN11 1.09 0.41 1.22 0 0.38 0.12 0.14 0.16 0.09 0.85 0.57 0.38 0.69 0 0
HIF1A 1.98 1.11 1.49 0 0.77 0.31 0.5 0.3 0.3 0.6 1.07 0.95 1.53 1.19 1.41
A3 B3 C3 S66 con1 con2 con3 con4 con5 A1 B1 C1 IBD1 S54 S78
Average Expression
2
1
0
F
Control
UC
CD4/AHR/DAPI
20μm
***
CD4+ AHR+cells in CD4+ T cells (%)
60
40
20
0
Control UC

Supplementary Figure 1. Single-cell RNA sequencing analysis identifies distinct T cell populations and AHR expression in colonic CD4+ T cells. (A) Dot plot showing the composition and sources of the sequenced samples. (B) Dot plot showing the expression of signature genes defining each cell cluster. (C) Violin plots displaying expression of lineage-identity genes for the annotation of the clusters of T cell subclusters (D) UMAP visualization of *CD3D, CD4, CD8A, CD8B* and reactive oxygen species pathway genes across the T cell subclusters. (E) Expression levels of *AHR, PTPN11,* and *HIF1A* of T cells in T/NK Cells of control and IBD samples. (F) The expression of AHR and CD4 in colon tissue in control and DSS-induced colitis mice, as assessed by Immunofluorescence staining. Data are presented as mean ± SD. UC, DSS- induced colitis *$P < 0.05$, **$P < 0.01$, ***$P < 0.001$

A

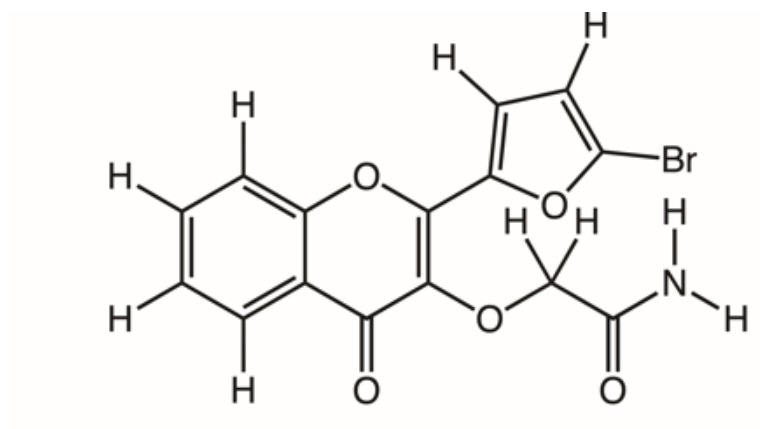

CB7993113

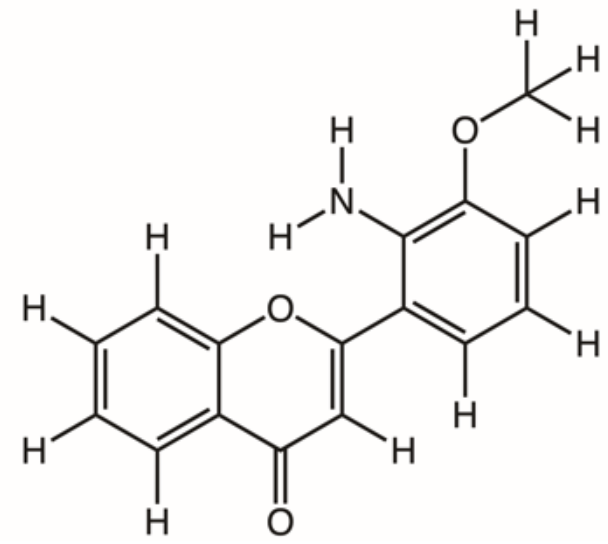

PD98059

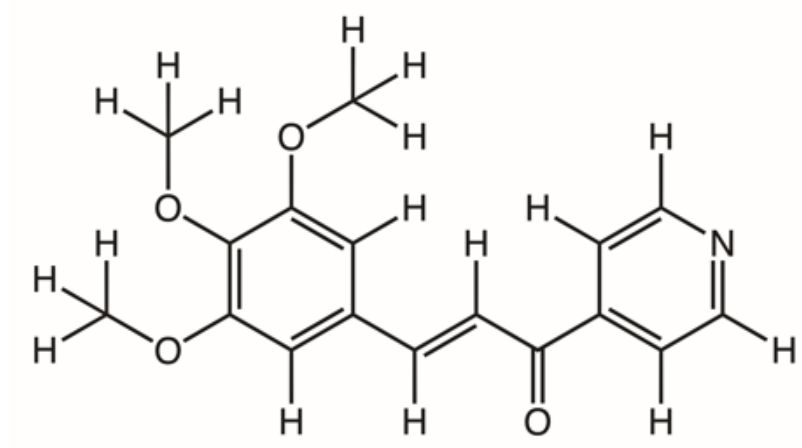

CYP1A1 inhibitor 8a

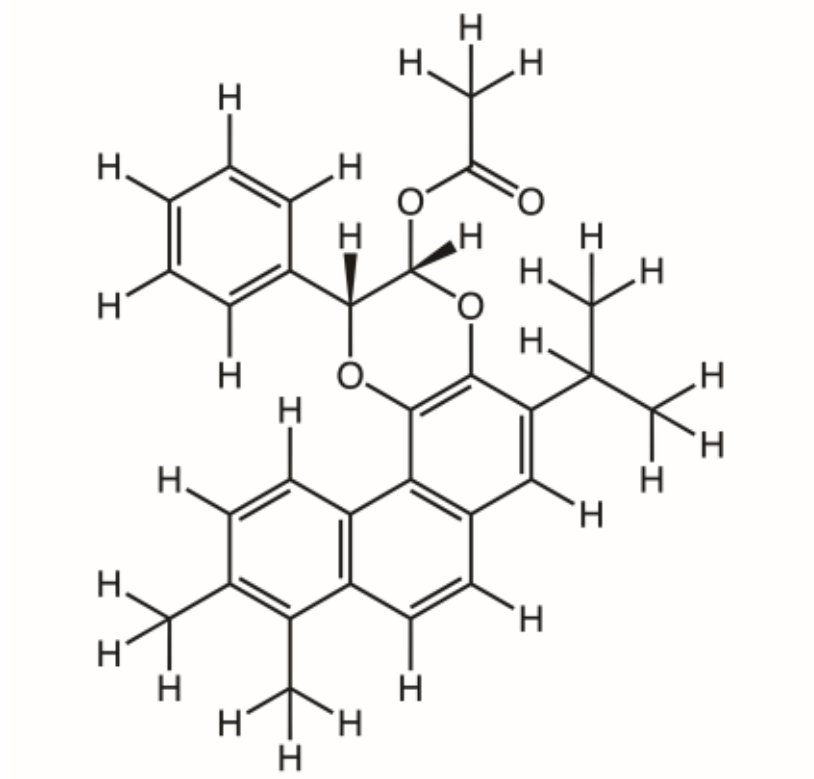

(2R,3S)-PD-1/PD-L1-IN-38

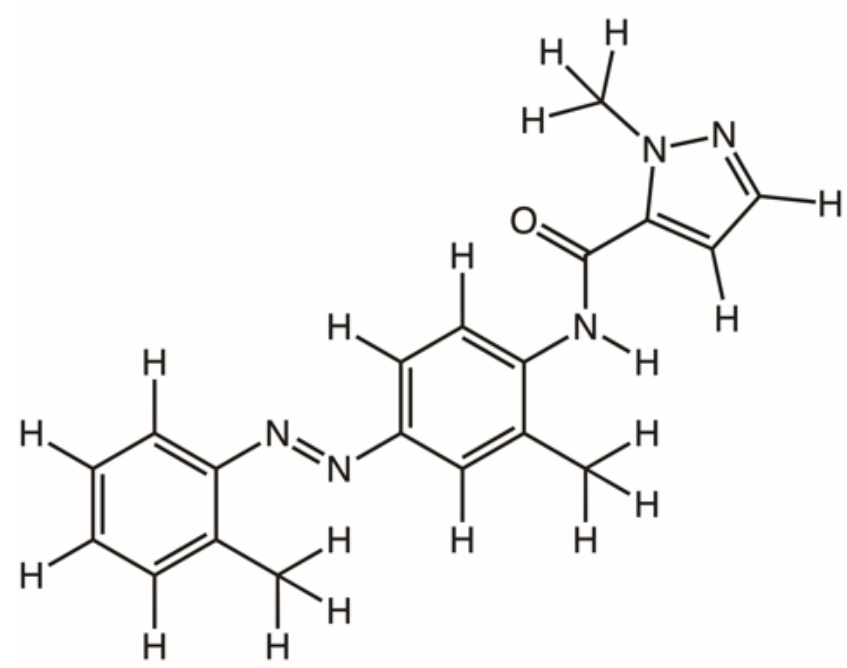

CH223191

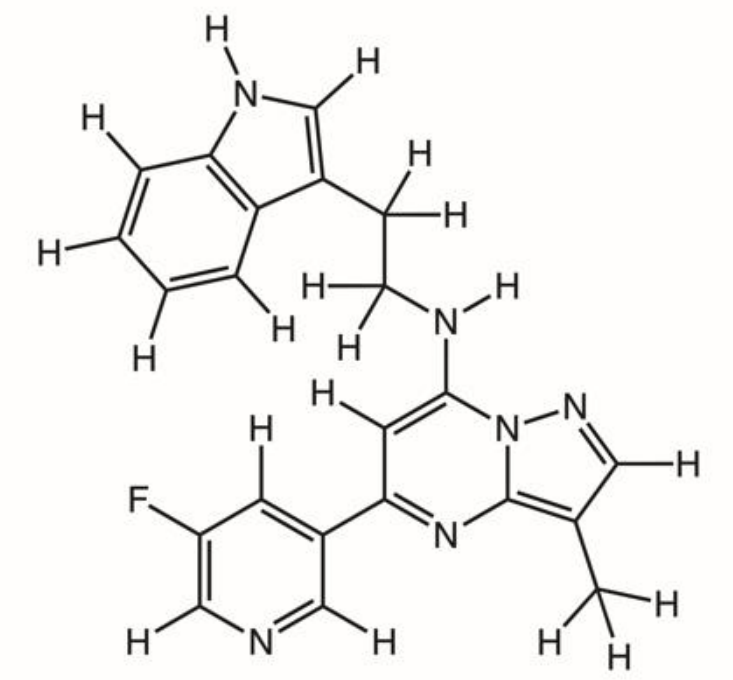

KYN-101

B

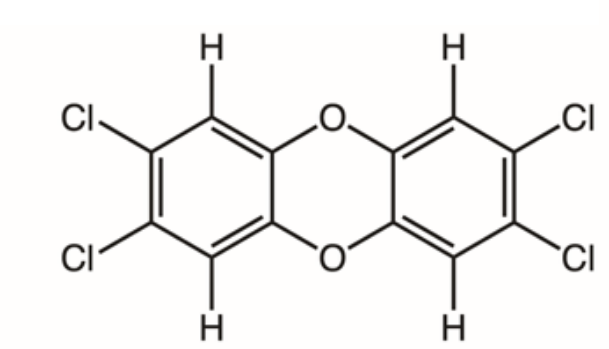

TCDD

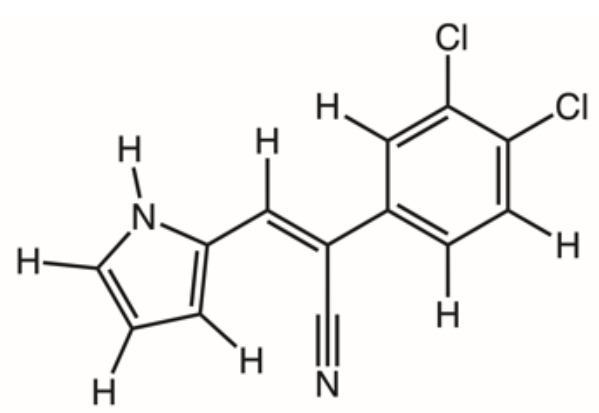

ANI-7

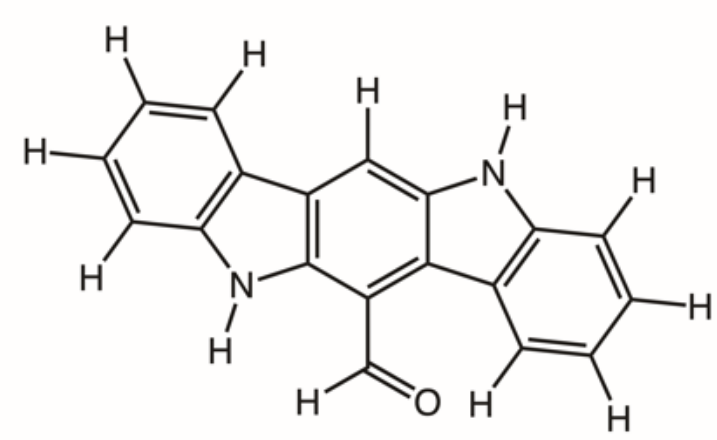

FICZ

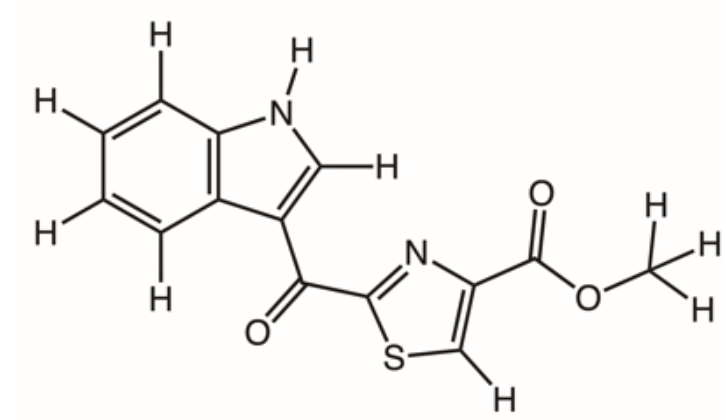

Pifithrin-α hydrobromide

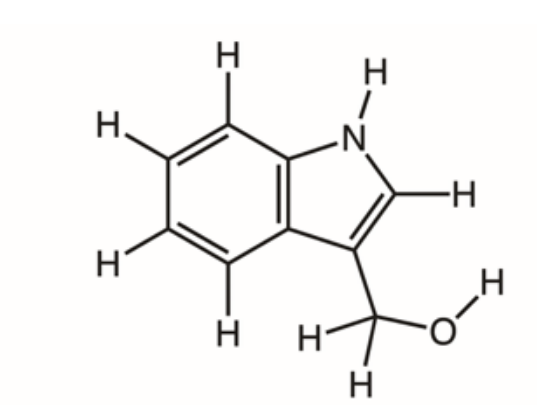

Indole-3-carbinol

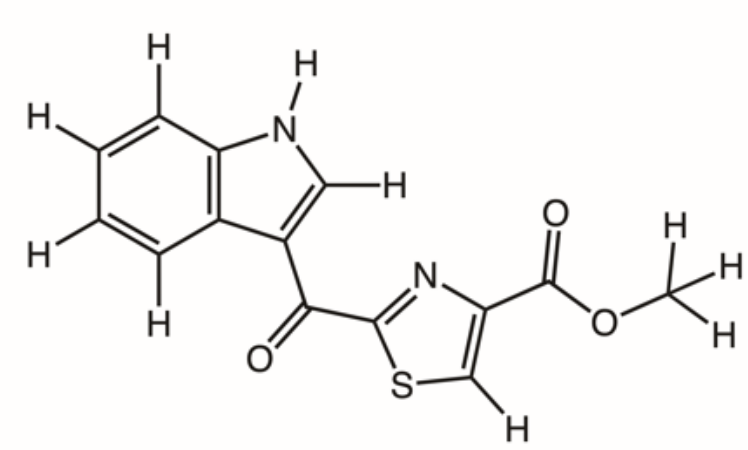

ITE

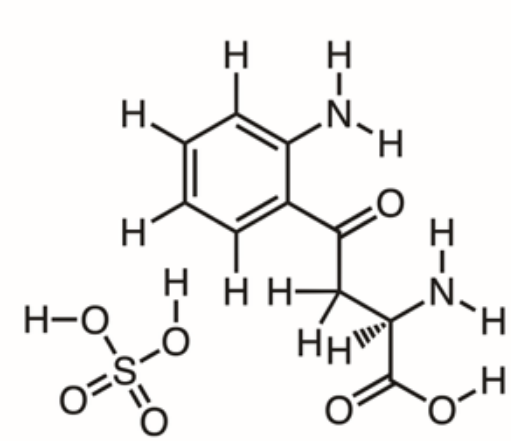

L-Kynurenine sulfate

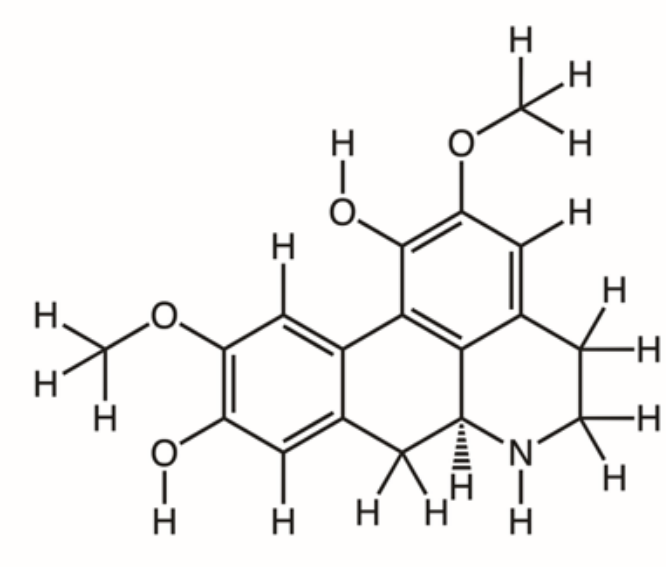

Norisoboldine

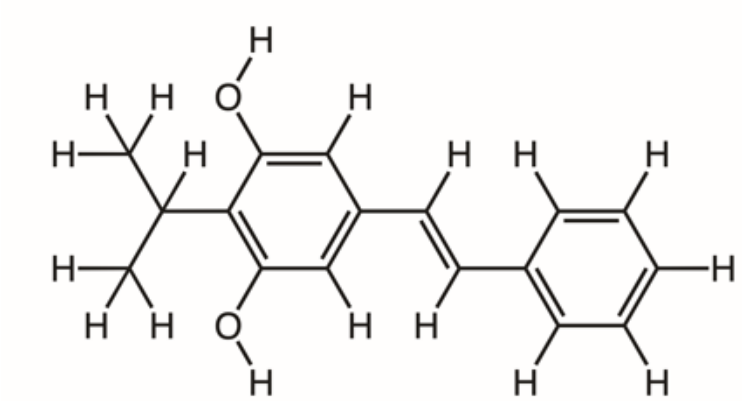

Tapinarof

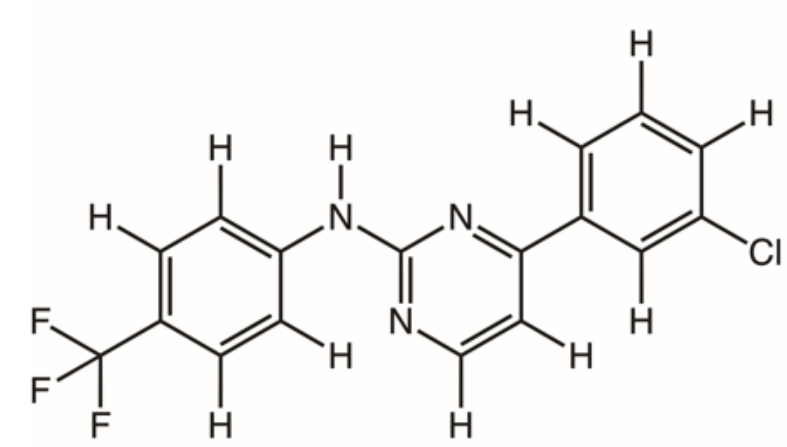

VAF347

Supplementary Figure 2. Pharmacological modulators of AHR signaling including the list of AHR antagonists and agonists (A) The molecular structure of AHR antagonist (B) The molecular structure of AHR agonists. These chemical structures were created by ChemDraw 22.2.

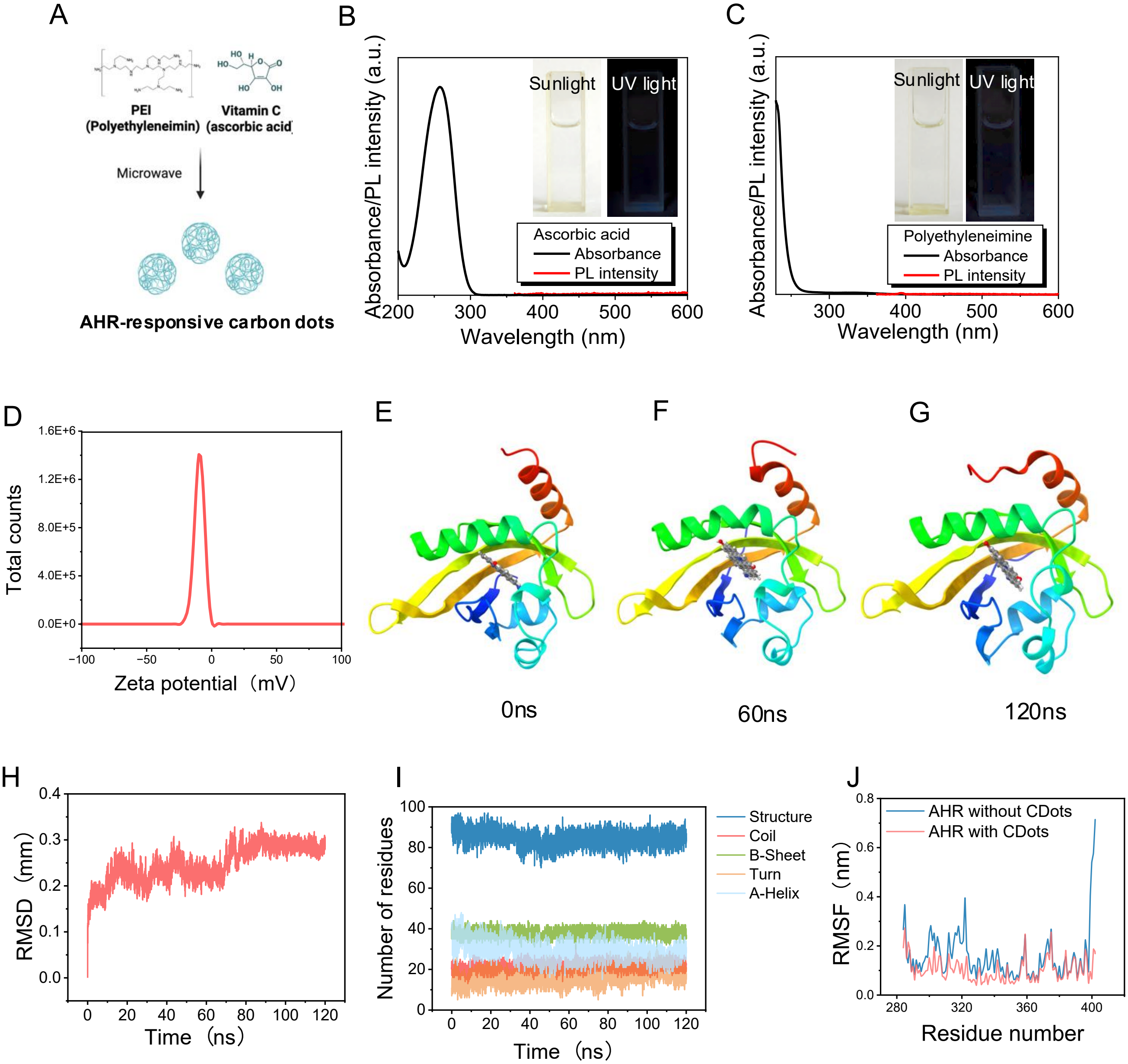

Supplementary Figure 3. Synthesis and molecular dynamics simulations of carbon dots with AHR. (A) The synthesis process of AHR-responsive carbon dots. This schematic illustration was created by BioRender. (B-C). The optical characteristics of the raw materials used for carbon dots synthesis under sunlight (left) and UV light (right), respectively. (B) Ascorbic acid, (C) Polyethyleneimine. (D) Zeta potential distribution of carbon dots, showing an average zeta potential of −9.529 mV.(E-G) Representative snapshots of the AHR and carbon dots complex extracted from molecular dynamics simulations at 0 ns (initial binding, E) ,60 ns (intermediate relaxation, F) and 120ns(equilibrium, G).Secondary structures are rendered as ribbons and colored by type: α-helices (cyan), 3-10 helices (blue), β-sheets (green), turns (orange), and coils (red). (H) The stability of AHR and carbon dots complex analyzed by root-mean-square deviation (RMSD). (I) Numbers of residues in diverse secondary structures , structures(blue), Coil (red),B-Sheet (green), Turn (orange), and A-Helix (cyan) (J) Root-mean-square fluctuation values of AHR and the AHR with carbon dots complex. Cdots, carbon dots.

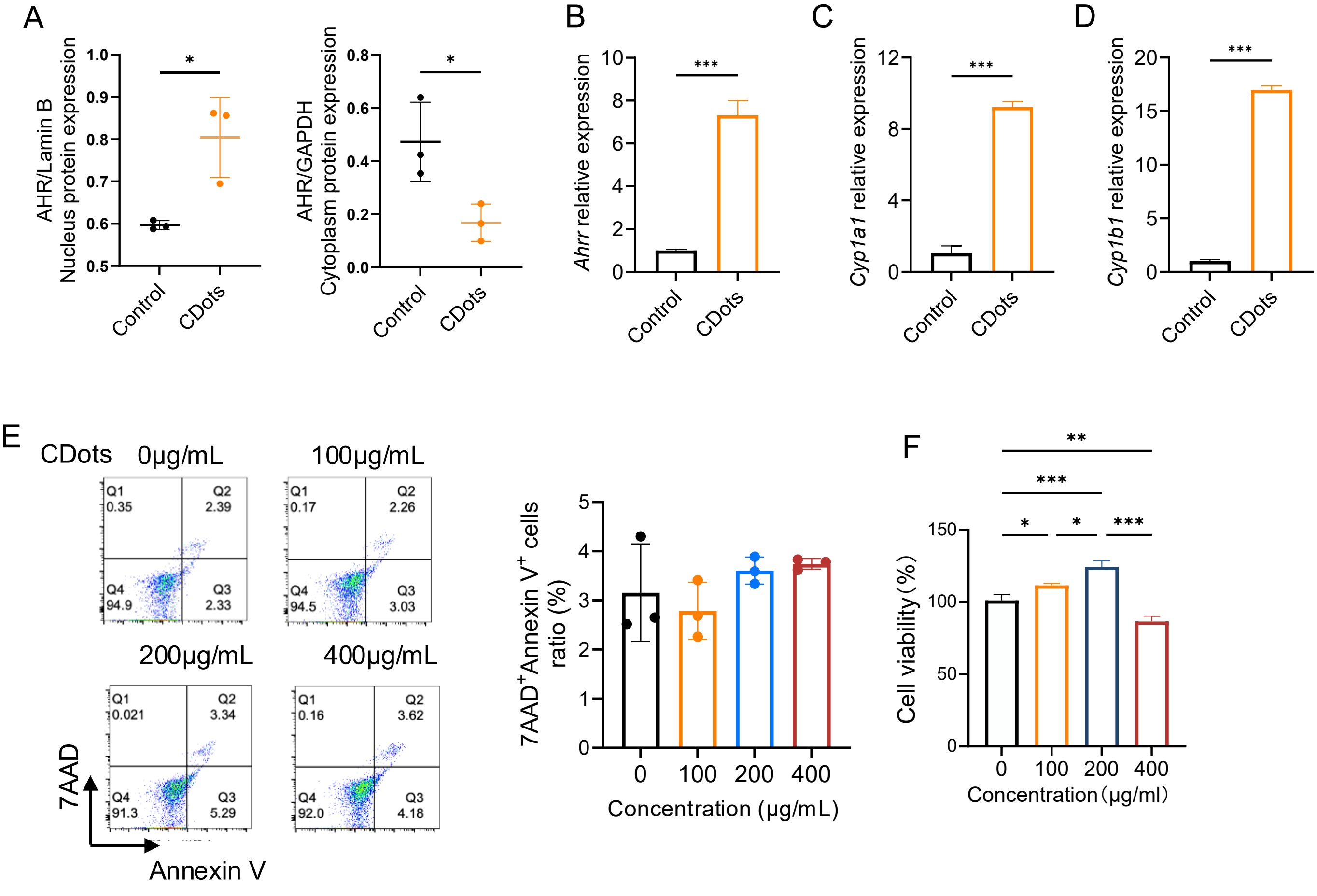

Supplementary Figure 4. AHR activation and in vitro biosafety evaluation of carbon dots. (A) Quantification of the nuclear/cytoplasmic AHR protein ratio in control and carbon dot-treated CD4$^+$ T cells. (B-D) The gene expression of *Ahrr*，*Cyp1a1* and *Cyp1b1* in control and carbon dot treated CD4$^+$ T cells under Treg cell polarization induction condition analyzed by RT-qPCR. (E) Apoptosis assay of T cells in control and 100, 200, and 400 μg/mL carbon dot treated groups, as assessed by Annexin V and 7AAD staining. (F) Cell viability of T cells in control and 100, 200, and 400 μg/mL carbon dot treated groups, as assessed by the CCK-8. Data are presented as mean±SD. CDots, carbon dots. *$P < 0.05$, **$P < 0.01$, ***$P < 0.001$

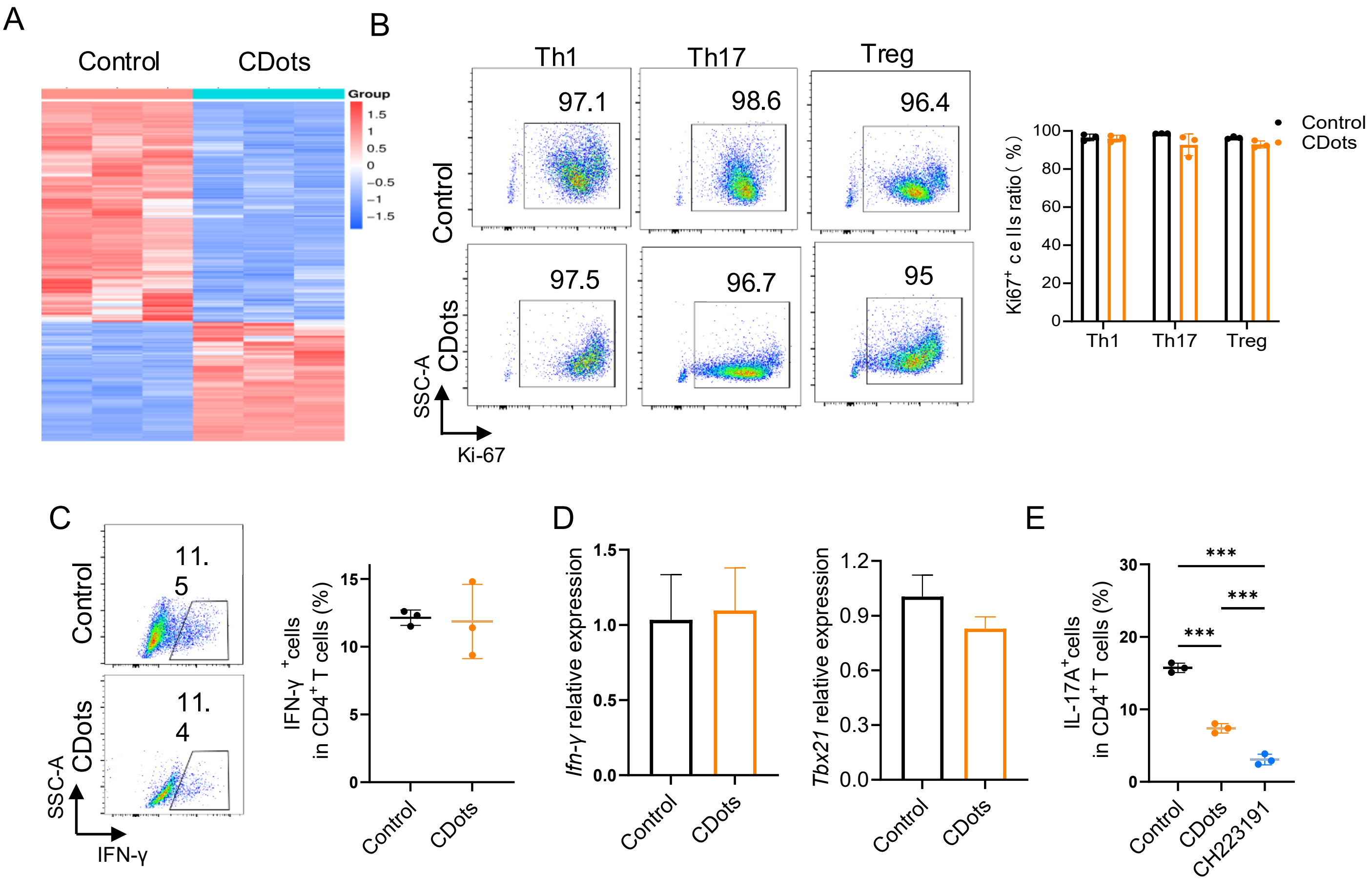

Supplementary Figure 5. Effects of carbon dots on T-cell proliferation and Th1/Th17 differentiation.(A) The different genes in control and carbon dot treated T cells analyzed by RNA-seq (Fold change >2 and p<0.05). (B) The proliferation rate of Th1, Th17 and Treg cells with or without carbon dot treatment, as assessed by Ki-67 staining. (C) IFN- γ^{+} Th1 cells ratio in $CD4^{+}$ T cells between control and carbon dot treated groups under Th1 cell polarization induction condition, as assessed by flow cytometry. (D) The gene expression of *Ifn-γ* and *Tbx21* in control and carbon dot treated CD4+ T cells under Th1 cell polarization induction condition analyzed by RT-qPCR. (E) The ratio of IL-$17A^{+}$ Th17 cells in control and carbon dot treated groups under Th17-polarizing conditions with or without AHR antagonist CH223191 treatment. Data are presented as mean±SD. CDots, carbon dots. *P < 0.05, **P < 0.01, ***P < 0.001

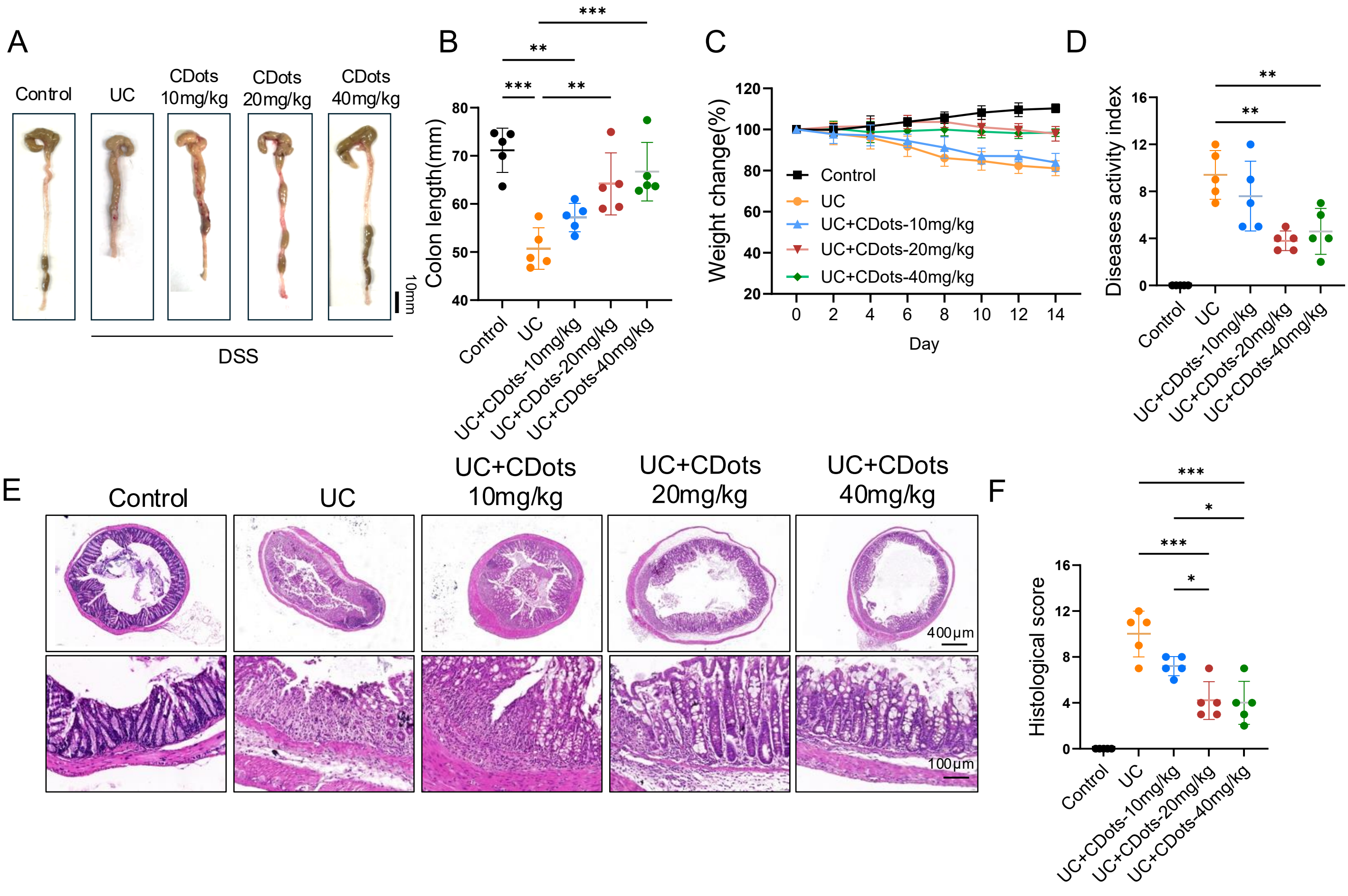

Supplementary Figure 6. Therapeutic effects of carbon dots at different doses in DSS-induced colitis mice.(A) Representative images of colon of control and DSS-induced colitis mice with or without carbon dot treatment at the indicated doses(10, 20, and 40 mg kg^{-1}). (B) The quantification of colon length in control and DSS-induced colitis mice with or without carbon dot treatment. (C) The body weight change in control and DSS-induced colitis mice with or without carbon dot treatment (D) The disease activity index (DAI) scores of controls and DSS-induced colitis mice with or without carbon dot treatment. (E) The histological structure of colons in control and DSS-induced colitis mice with or without carbon dot treatment, as assessed by H&E staining. (F) The histological scores in control and DSS-induced colitis mice with or without carbon dot treatment. Data are presented as mean ± SD. $*P < 0.05$, $**P < 0.01$, $***P < 0.001$.

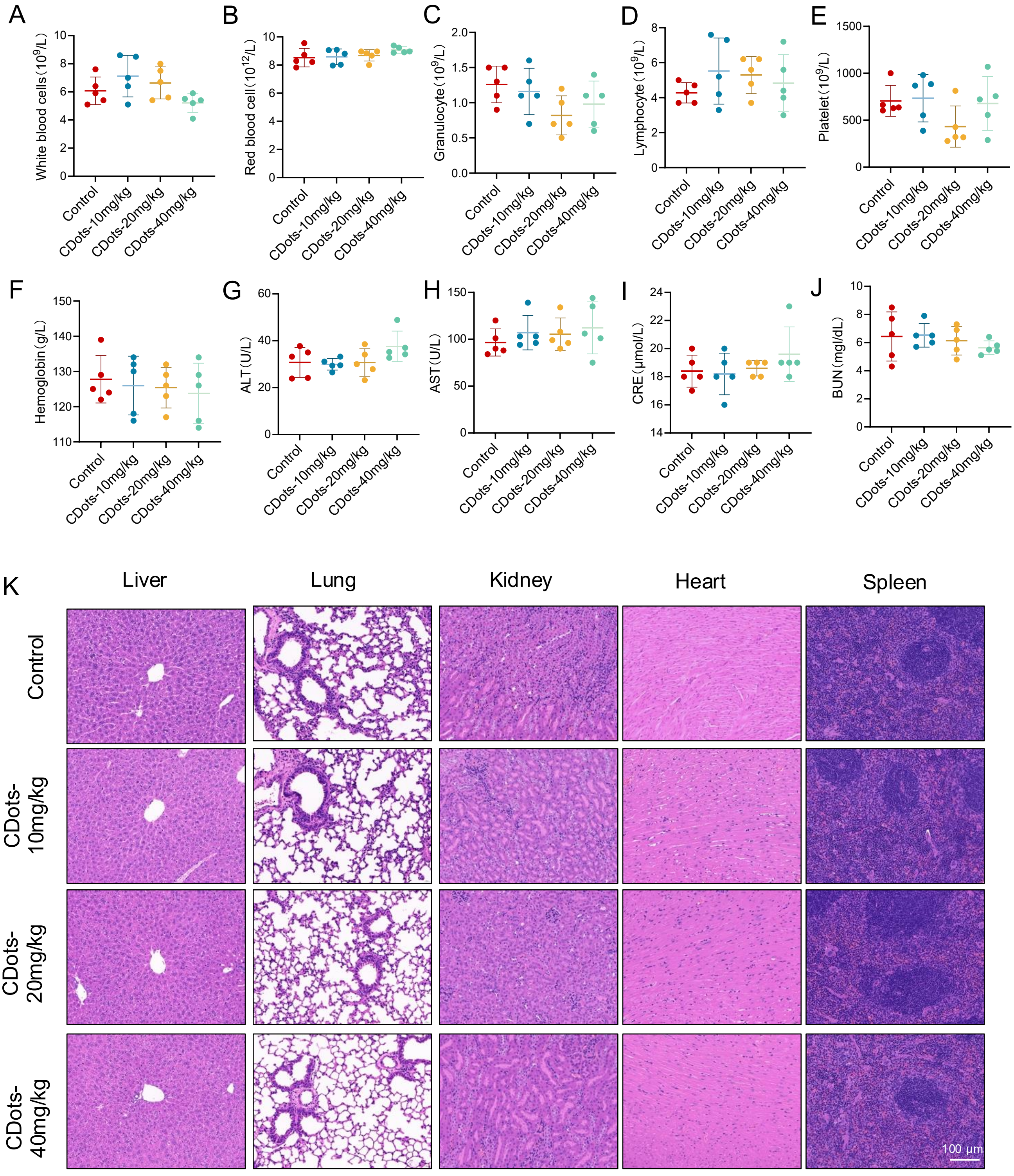

Supplementary Figure 7. *In vivo* biosafety evaluation of carbon dots at different doses. (A–F) Routine hematological parameters of mice treated with carbon dots at the indicated doses (10, 20, and 40 mg kg^{-1}), including white blood cell (A), red blood cell count (B), granulocyte count (C), lymphocyte count (D), platelet count (E), and hemoglobin concentration (F). (G–J) Serum biochemical parameters, including alanine aminotransferase (G), aspartate aminotransferase (H), creatinine (I), and blood urea nitrogen (J). (K)The histological structure of liver, lung, kidney, heart, and spleen from control mice and mice treated with carbon dots at the indicated doses , as assessed by H&E staining. Scale bar, 100 μm; Data are presented as mean ± SD. CDots, carbon dots; WBC, white blood cell count; RBC, red blood cell count; GRA, granulocyte count; Lymph, lymphocyte count; PLT, platelet count; HGB, hemoglobin; ALT, alanine aminotransferase; AST, aspartate aminotransferase; CRE, creatinine; BUN, blood urea nitrogen. *$P < 0.05$, **$P < 0.01$, ***$P < 0.001$n = 5 mice per group.

A

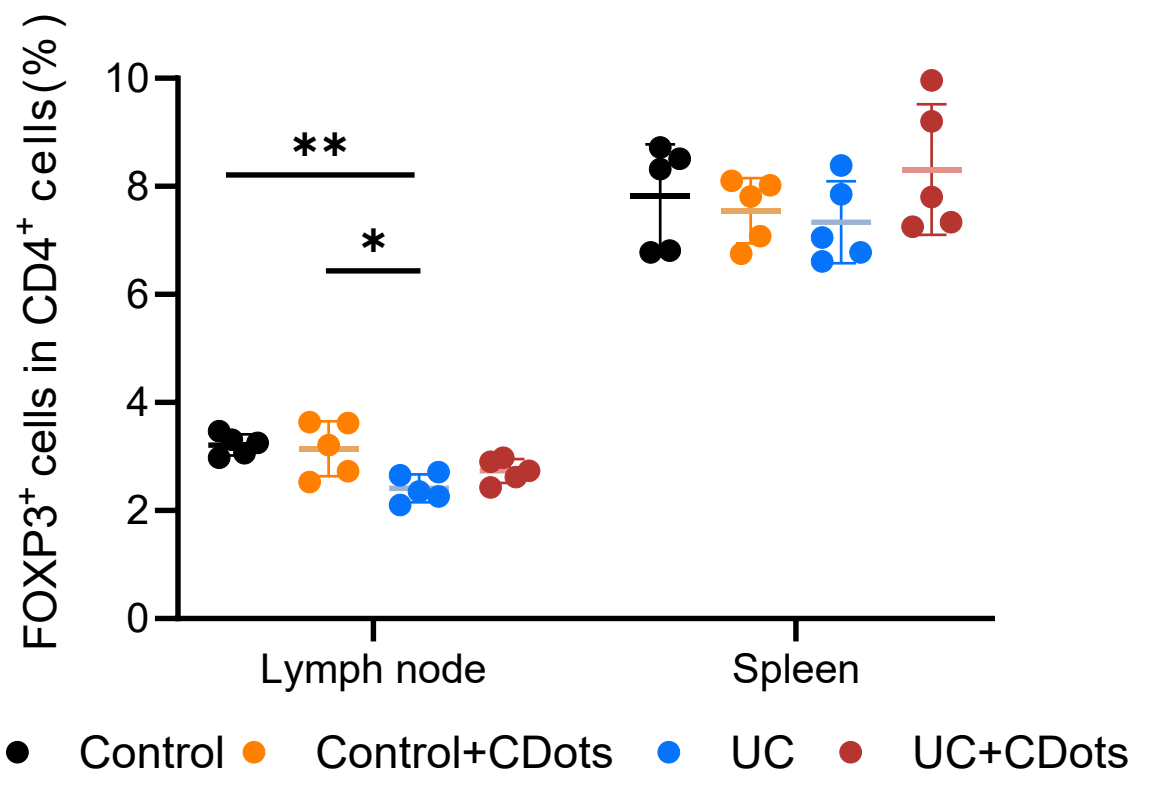

Supplementary Figure 8. Effects of carbon dots on systemic Treg populations. (A) The proportions of FOXP3$^+$ Treg cells in lymph nodes and spleens in control and DSS-induced colitis mice with or without carbon dots. Data are presented as mean ± SD. CDots, carbon dots; UC, DSS- induced colitis. *P < 0.05, **P < 0.01, ***P < 0.001

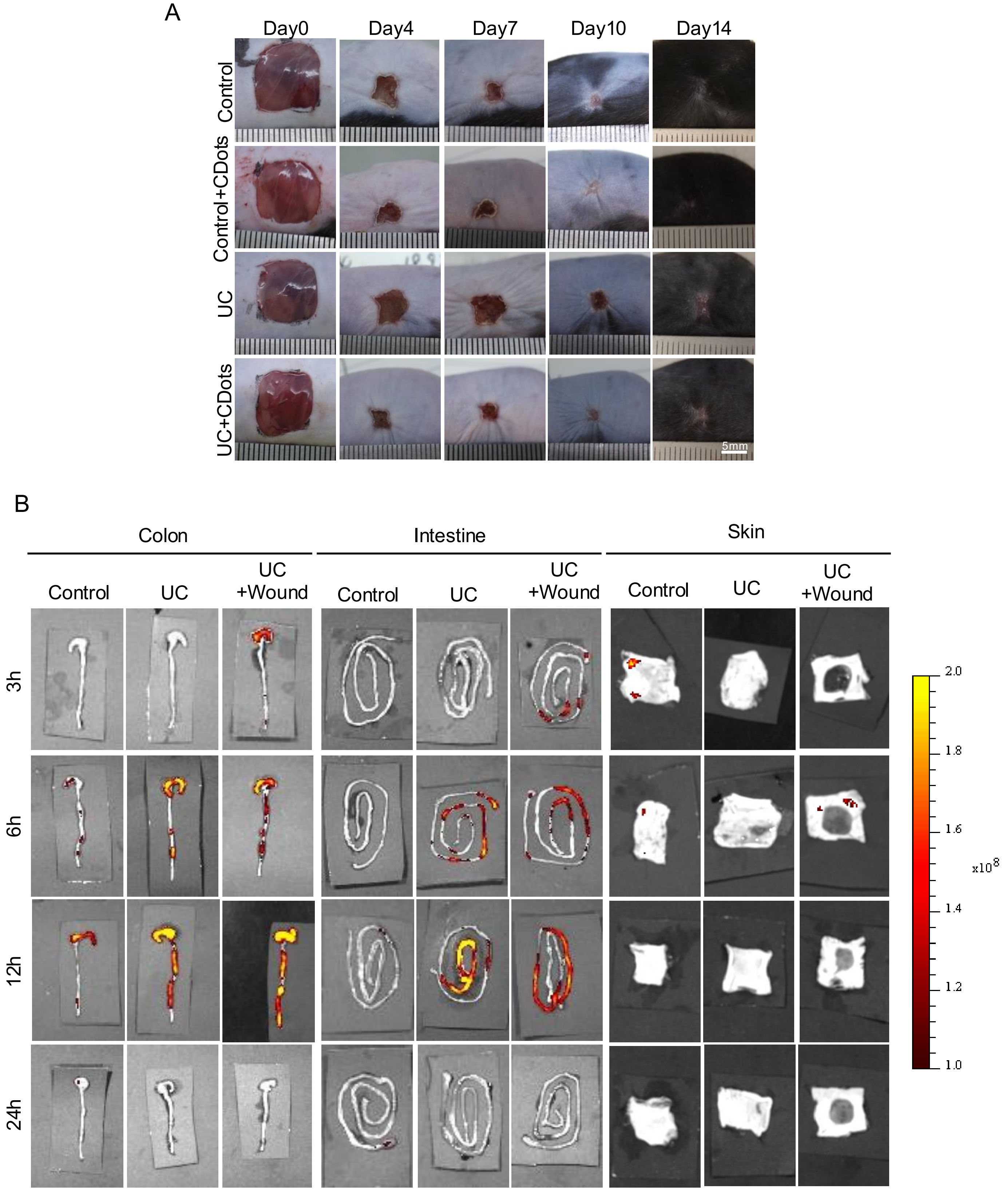

Supplementary Figure 9. AHR-responsive carbon dots promote wound healing. (A) The representative images of skin lesions at days 0, 4, 7, 10, and 14 post-injury in control and DSS-induced colitis mice with or without carbon dot treatment. (B) The accumulation of carbon dots in colon, intestine and skin at 3, 6, 12 and 24 hours post carbon dots injection in control, colitis and colitis combined skin wound mice. Cdots, carbon dots; UC, DSS- induced colitis.

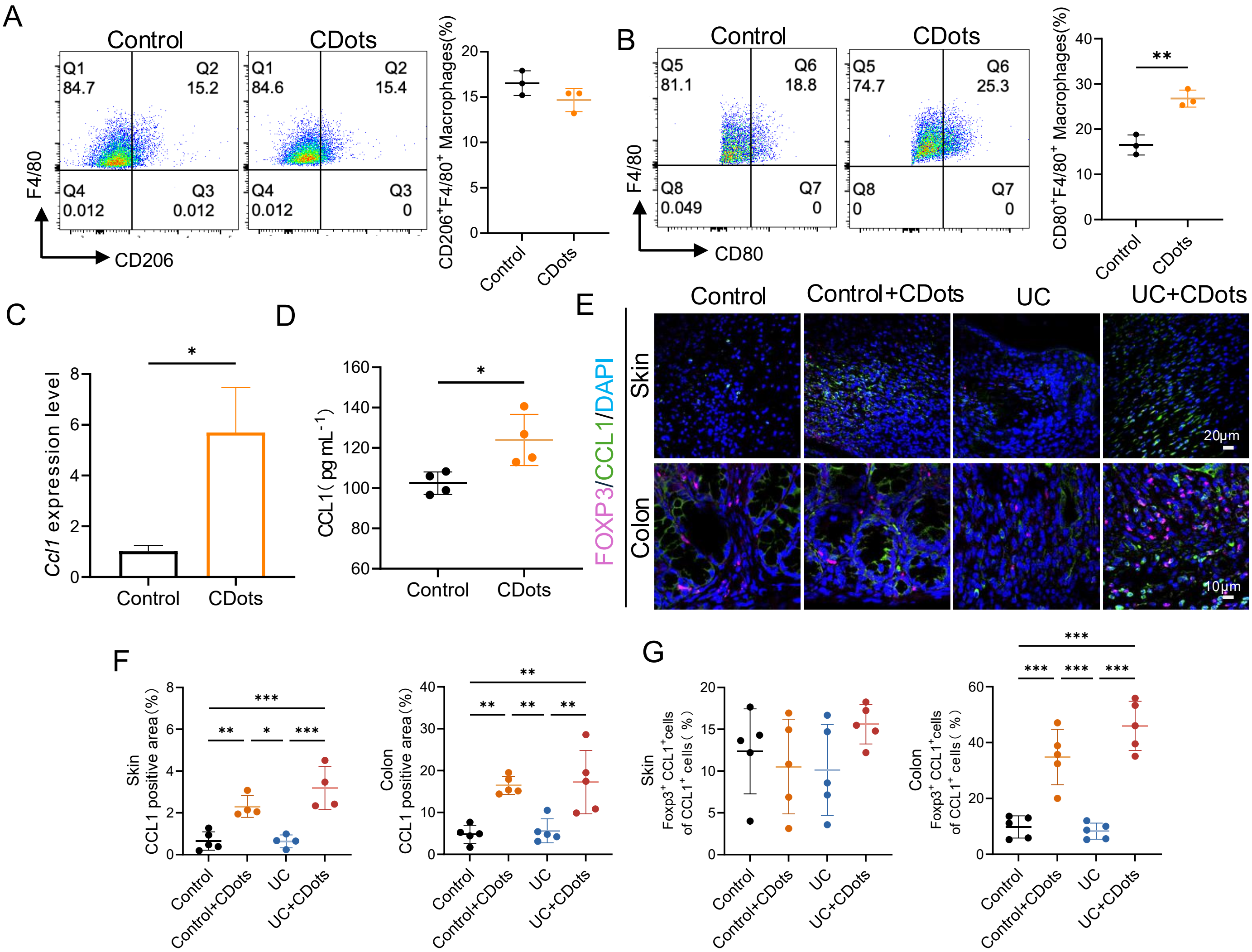

Supplementary Figure 10. AHR-responsive carbon dots enhance Treg-associated CCL1 expression.(A)The ratio of $CD206^+$ and $F4/80^+$ double-positive M2 macrophages in control and carbon dot treated BMDMs, assessed by flow cytometry. (B)The ratio of $CD80^+$ and $F4/80^+$ double-positive M1 macrophages in control and carbon dot treated BMDMs, assessed by flow cytometry.(C)The gene expression of *Ccl1* in control and carbon dot treated Treg cells. (D) Supernatant CCL1 levels in control and carbon dot treated Treg cells , as assessed by ELISA. (E) Representative immunofluorescence images showing CCL1 and FOXP3 expression in skin lesions and colon tissues 7 days after injury in control and DSS-induced colitis mice with or without carbon dot treatment. (F) Quantification of CCL1 expression in skin lesions and colon tissues. (G)Quantification of the proportion of $FOXP3^+CCL1^+$ cells among total $CCL1^+$cells in skin lesions and colon tissues. Data are presented as mean ± SD. CDots, carbon dots. UC, DSS induced colitis.*P < 0.05, **P < 0.01, ***P < 0.001.

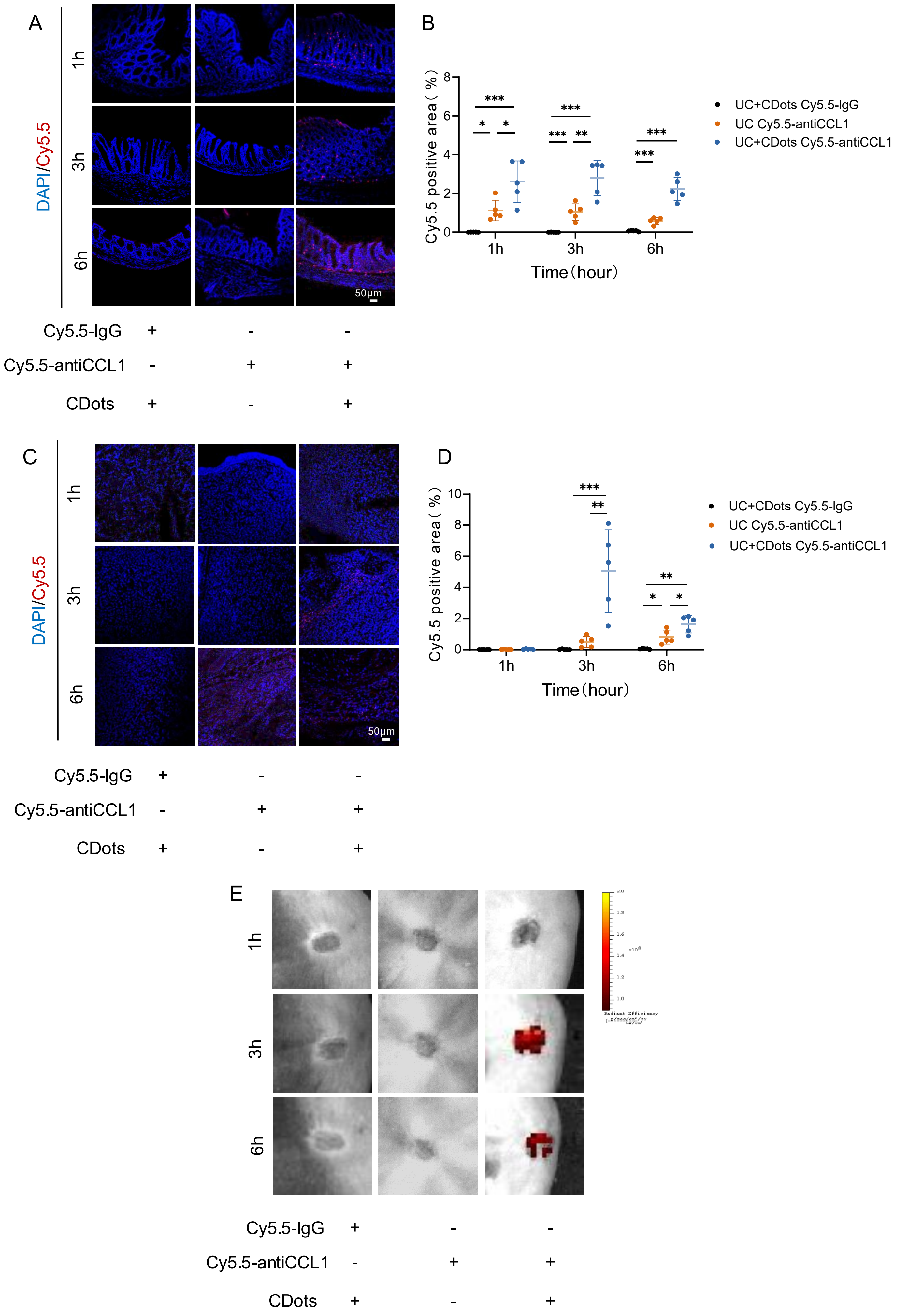
A
DAPI/Cy5.5
1h
3h
6h
50μm
Cy5.5-IgG + - -
Cy5.5-antiCCL1 - + +
CDots + - +
B
Cy5.5 positive area (%)
Time (hour)
1h
3h
6h
UC+CDots Cy5.5-IgG
UC Cy5.5-antiCCL1
UC+CDots Cy5.5-antiCCL1
C
DAPI/Cy5.5
1h
3h
6h
50μm
Cy5.5-IgG + - -
Cy5.5-antiCCL1 - + +
CDots + - +
D
Cy5.5 positive area (%)
Time (hour)
1h
3h
6h
UC+CDots Cy5.5-IgG
UC Cy5.5-antiCCL1
UC+CDots Cy5.5-antiCCL1
E
1h
3h
6h
Radiant Efficiency
Cy5.5-IgG + - -
Cy5.5-antiCCL1 - + +
CDots + - +

Supplementary Figure 11. AHR-responsive carbon dots promote the colon-derived CCL1 to distal skin wounds. (A,B) Representative immunofluorescence images and quantification of Cy5.5 fluorescence in colon tissues at 1, 3 and 6 h after CCL1-antibody or IgG probe intramural injection in DSS-induced colitis mice with or without carbon dot treatment. (C,D) Representative immunofluorescence images and quantification of Cy5.5 fluorescence in skin wound at 1, 3 and 6 h after CCL1-antibody or IgG probe intramural injection in DSS-induced colitis mice with or without carbon dot treatment. (E) *In vivo* fluorescence imaging of the skin wound at 1, 3 and 6 h after CCL1-antibody or IgG probe intramural injection in DSS-induced colitis mice with or without carbon dot treatment. Data are presented as mean ± SD. CDots, carbon dots. UC, DSS induced colitis.*$P < 0.05$, **$P < 0.01$, ***$P < 0.001$.

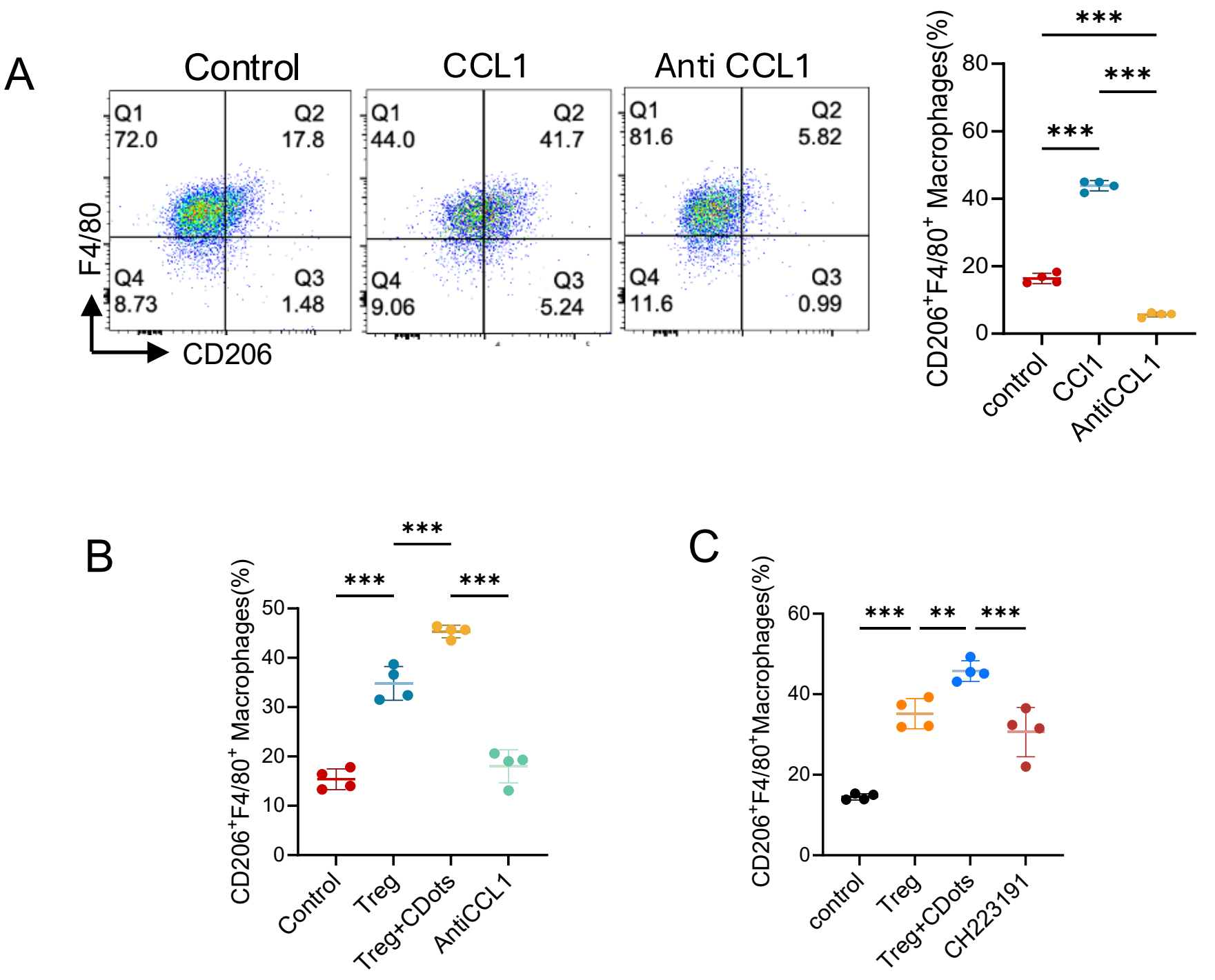

Supplementary Figure 12. AHR responsive Carbon dots promoted M2 macrophage polarization through Treg-derived CCL1.(A) The ratio of CD206$^+$ and F4/80$^+$ double-positive BMDMs treated with CCL1 or anti-CCL1, as assessed by flow cytometry.(B) The ratio of CD206$^+$ and F4/80$^+$ double-positive BMDMs in control，Treg cells and carbon dot treated-Treg cells co-culture groups with or without CCL1 neutralizing antibody administration, assessed by flow cytometry (C) The ratio of CD206$^+$ and F4/80$^+$ double-positive BMDMs in control，Treg cells and carbon dot treated-Treg cells co-culture groups with or without AHR antagonist CH2231913 administration, as assessed by flow cytometry. Data are presented as mean ± SD. Cdots, carbon dots. UC, DSS induced colitis.*P < 0.05, **P < 0.01, ***P < 0.001.

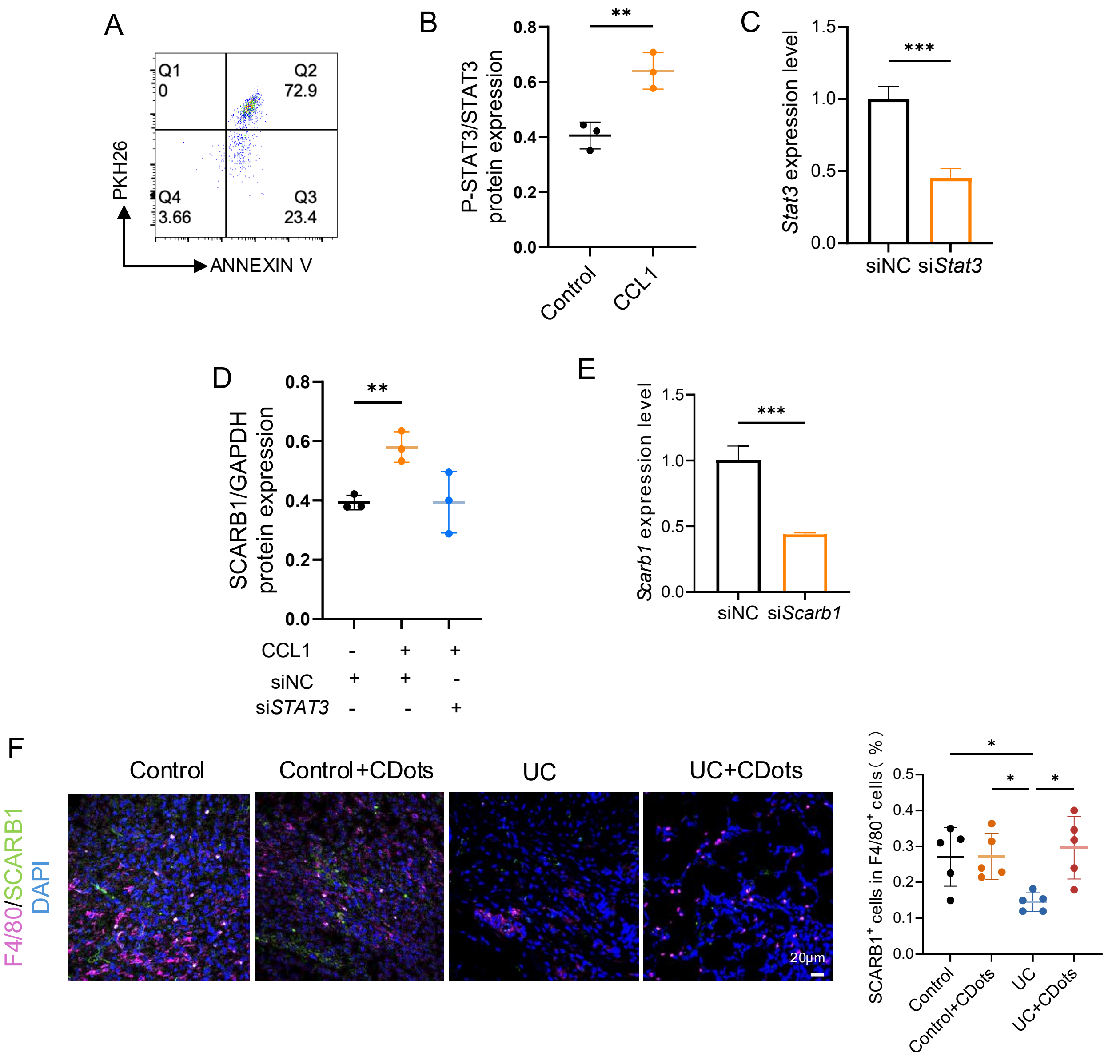

Supplementary Figure 13. AHR-responsive carbon dots increase SCARB1 expression in wound-associated macrophages. (A) The Annexin V-positive ratio of PKH26-labeled apoptotic neutrophils, as assessed by flow cytometry. (B) Quantification of phosphorylated STAT3 (p-STAT3) protein expression in BMDMs treated with or without CCL1. (C) The gene expression of *Stat3* in BMDMs in control and *Stat3* siRNA treated BMDMs, assessed by RT-qPCR. (D) Quantification of SCARB1 protein expression of BMDMs in control and STAT3 siRNA treated groups, with or without CCL1 administration. (E) The gene expression of *Scarb1* in BMDMs in control and *Scarb1* siRNA treated BMDMs, assessed by RT-qPCR. (F) Representative immunofluorescence images and quantification of SCARB1^{+} cells among F4/80^{+} macrophages in skin lesions in control and DSS-induced colitis mice with or without carbon dot treatment. Data are presented as mean ± SD. CDots, carbon dots. UC, DSS induced colitis.*P < 0.05, **P < 0.01, ***P < 0.001.

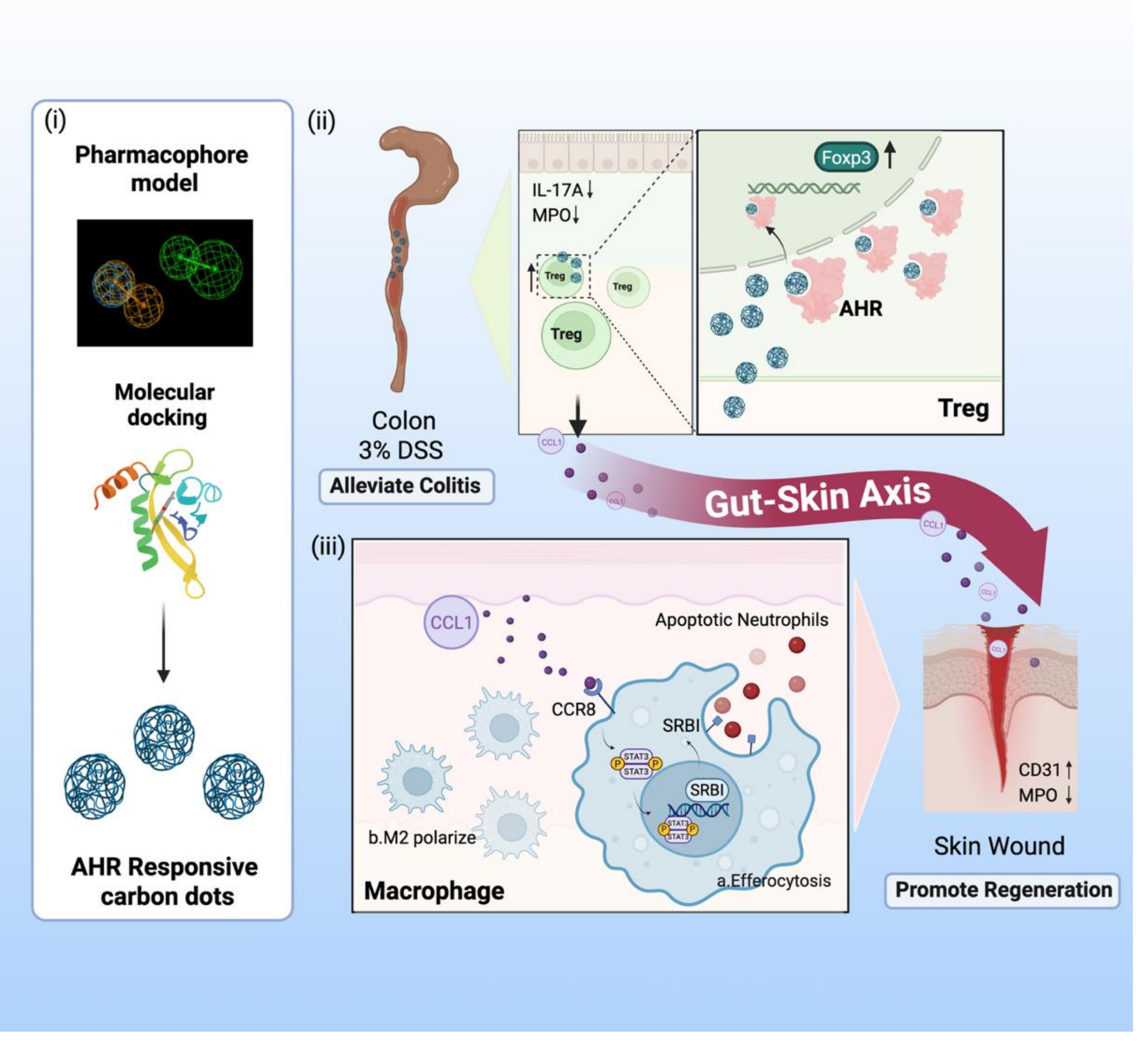

Supplementary Figure 14. Schematic illustration showed that AHR-responsive carbon dots promote cutaneous wound healing through gut-resident regulatory T cells induced macrophage efferocytosis. AHR-responsive carbon dots promote cutaneous wound healing and colonic mucosal repair through gut-resident Treg-mediated macrophage efferocytosis. (i) Guided by pharmacophore modeling and molecular docking, AHR-responsive carbon dots were synthesized. (ii, iii) These AHR-responsive carbon dots directly bind to AHR and expand gut-resident Treg cells. CCL1 secreted by these tissue-resident Treg cells enhances macrophage efferocytosis and establishes a pro-regenerative niche, thereby accelerating both skin wound healing and colonic mucosal repair via the gut–skin axis. This schematic illustration was created by BioRender.

Fig.2I

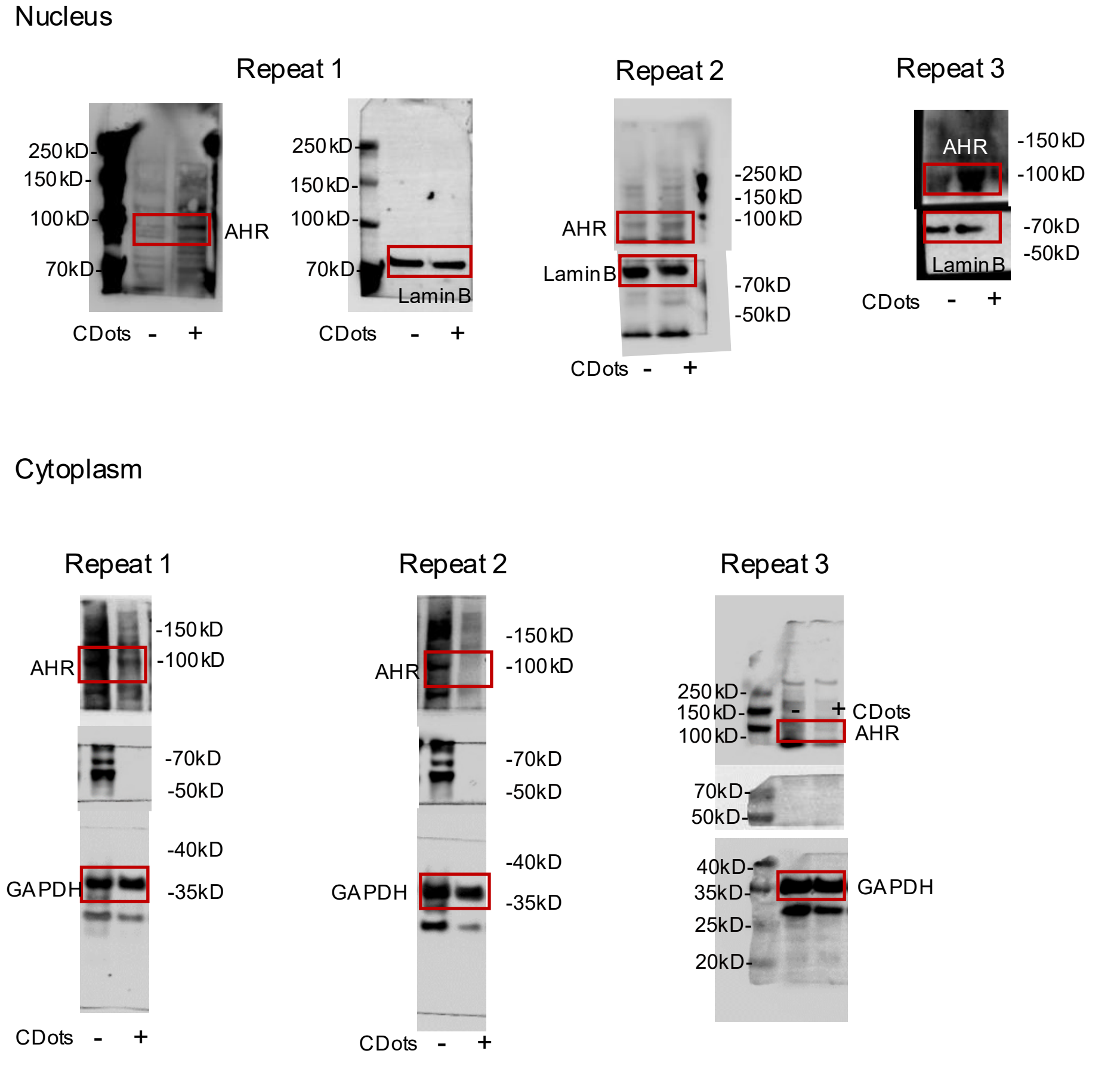

Supplementary Figure 15. Uncropped western blot images and repeated experiments corresponding to Figure. 2I.

Fig.6M

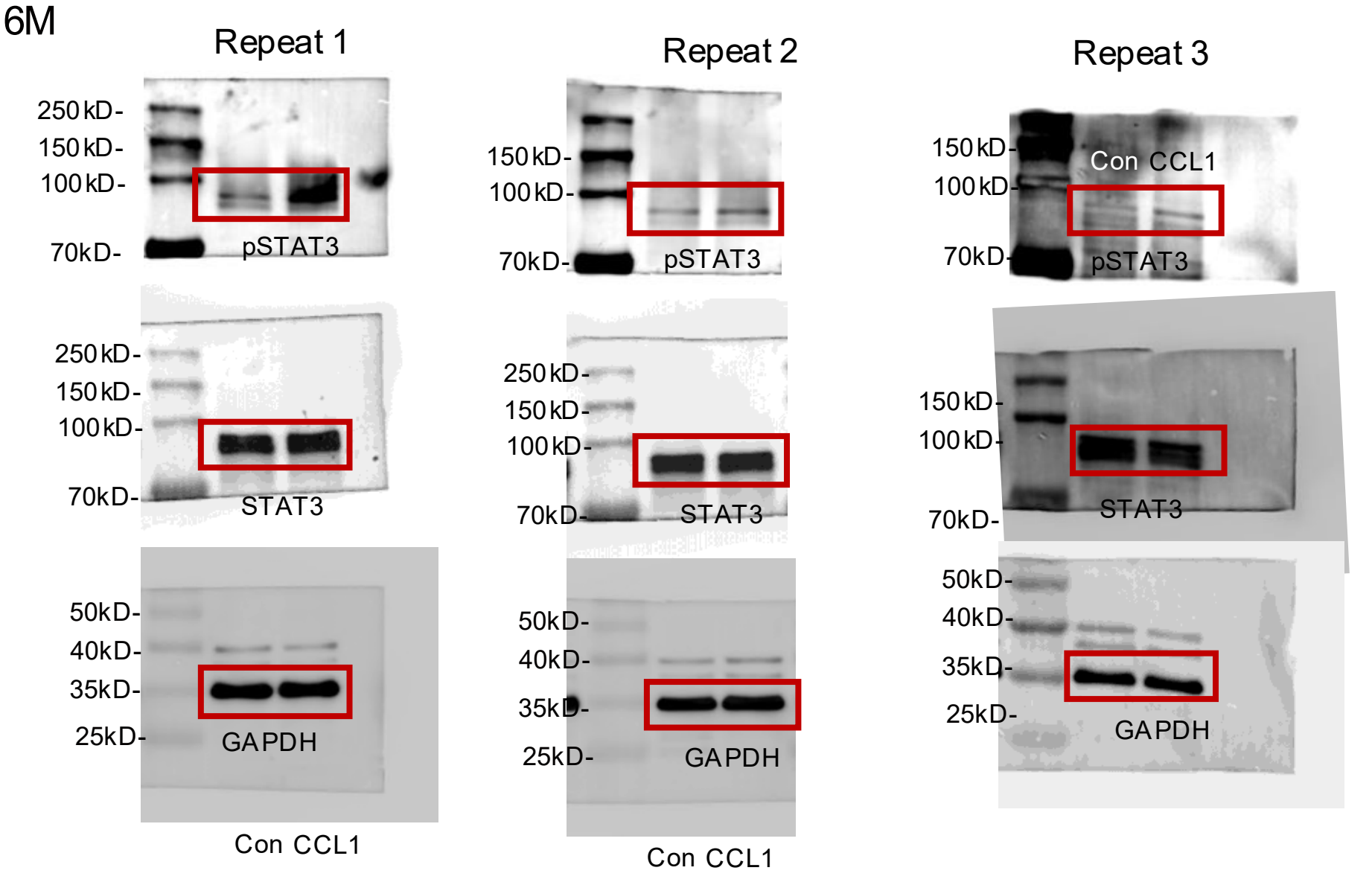

Fig.6P

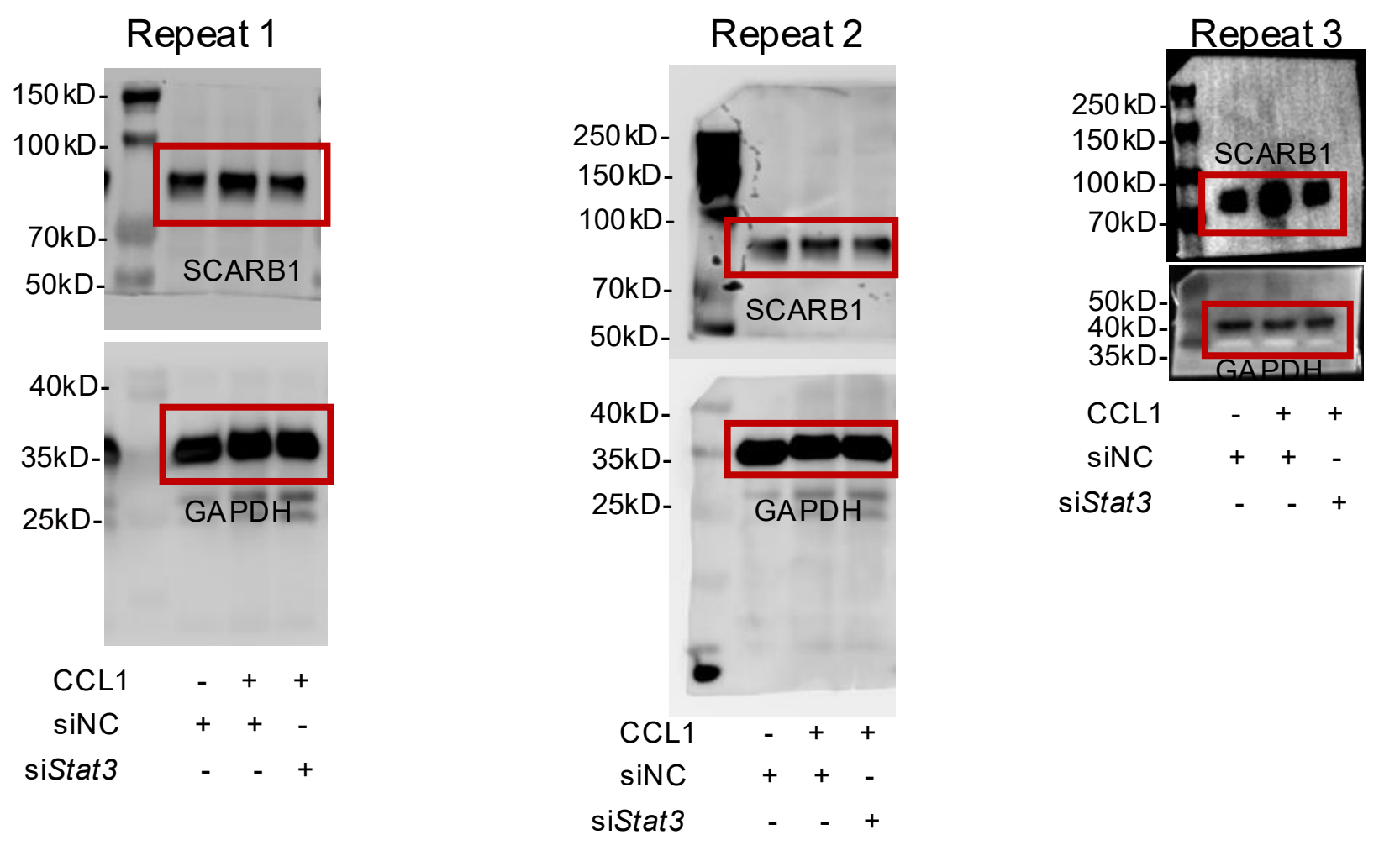

Supplementary Figure 16. Uncropped western blot images and repeated experiments corresponding to Figure. 6M, 6P.

Supplementary Table1: Antibodies and cytokines

Antibodies		
Reagent	Catalog No.	Company
Anti-mouse CD4	17-0042-83	eBioscience, San Diego, CA, USA
Anti-mouse CD25	25-0251-82	eBioscience, San Diego, CA, USA
Anti-mouse CD62L	47-0621-82	eBioscience, San Diego, CA, USA
Anti-mouse CD44	48-0441-82	eBioscience, San Diego, CA, USA
Anti-mouse IL-17A	48-7177-82	eBioscience, San Diego, CA, USA
Anti-mouse IFN-γ	48-7311-82	eBioscience, San Diego, CA, USA
Anti-mouse Foxp3	48-5773-82	eBioscience, San Diego, CA, USA
Anti-mouse Ki67	556027	BD Biosciences, Franklin Lakes, NJ, USA
Anti-mouse AhR	MA1-513	Thermo Fisher, Waltham, MA, USA
Anti-mouse eFluor™ 570 anti-Foxp3	41-5773-82	Thermo Fisher, Waltham, MA, USA
Anti-mouse F4/80	14-4801-85	Thermo Fisher, Waltham, MA, USA
Fixable Viability Dye	65-0864-18	Thermo Fisher, Waltham, MA, USA
Anti-mouse IL-17A	A26163	Proteintech, Rosemont, IL, USA
Anti-mouse iNOS	22226-1-AP	Proteintech, Rosemont, IL, USA
Anti-mouse GAPDH	1E6D9	Proteintech, Rosemont, IL, USA
Anti-mouse CCL1	98502-2-RR	Proteintech, Rosemont, IL, USA
Anti-mouse CCL1	AF485	R&D Systems, Minneapolis, MN, USA
Anti-mouse MPO	AF3667	R&D Systems, Minneapolis, MN, USA
Anti-mouse CD206	ab64693	Abcam, Cambridge, MA, USA
Anti-mouse CD206	141705	BioLegend, San Diego, CA, USA
Anti-mouse CD80	560526	BD Biosciences, Franklin Lakes, NJ, USA
Anti-mouse SCARB1	ab217318	Abcam, Cambridge, MA, USA
Anti-mouse Ly6G	sc-53515	Santa Cruz Biotechnology, Dallas, TX, USA
Anti-mouse STAT3	#9139	Cell Signaling Technology, Danvers, MA, USA
Anti-mouse Phospho-Stat3 (Tyr705)	#9145	Cell Signaling Technology, Danvers, MA, USA
Anti-mouse CD3	BE0002	Bio X Cell, West Lebanon, NH, USA
Anti-mouse CD28	BE0015	Bio X Cell, West Lebanon, NH, USA

Cytokines		
Reagent	Catalog No.	Company
Mouse IL-4	BE0045	Bio X Cell, West Lebanon, NH, USA
Mouse IL-2	402-ML	PeproTech, Rocky Hill, NJ, USA
Mouse IL-12	210-12	PeproTech, Rocky Hill, NJ, USA
Mouse IL-6	216-16	PeproTech, Rocky Hill, NJ, USA
Macrophage colony-stimulating factor (M-CSF)	315-02	PeproTech, Rocky Hill, NJ, USA
Human TGF-β	240-B	R&D Systems, Minneapolis, MN, USA

Supplementary Table 2. Primer sequences for RT-qPCR and siRNA

Genes		Primer sequence
Ahr	Forward	CTGGTTGTCACAGCAGATGCCT
Ahr	Reverse	CGGTCTTCTGTATGGATGAGCTC
Foxp3	Forward	CTGTGCGACTTAAGAAACAGAGTC
Foxp3	Reverse	CTGCCTCTCCCATTATCACTACC
Il17a	Forward	CAGACTACCTCAACCGTTCCAC
Il17a	Reverse	TCCAGCTTTCCCTCCGCATTGA
Tbx21	Forward	CCACCTGTTGTGGTCCAAGTTC
Tbx21	Reverse	CCACAAACATCCTGTAATGGCTTG
Rorc	Forward	GTGGAGTTTGCCAAGCGGCTTT
Rorc	Reverse	CCTGCACATTCTGACTAGGACG
Ifng	Forward	CAGCAACAGCAAGGCGAAAAAGG
Ifng	Reverse	TTTCCGCTTCCTGAGGCTGGAT
Scarb1	Forward	ACACCCGAATCCTCGCTGGAAT
Scarb1	Reverse	CCGTTGGCAAACAGAGTATCGG
Stat3	Forward	AGGAGTCTAACAACGGCAGCCT
Stat3	Reverse	GTGGTACACCTCAGTCTCGAAG
Ccl1	Forward	GCTTACGGTCTCCAATAGCTGC
Ccl1	Reverse	GCTTTCTCTACCTTTGTTCAGCC
β-Actin	Forward	GGCTGTATTCCCCTCCATCG
β-Actin	Reverse	CCAGTTGGTAACAATGCCATGT
si*Scarb1*	Forward	CCAUGAAGCUGACCUACA
si*Scarb1*	Reverse	UGUAGGUCAGCUUCAUUGG
si*Stat3*	Forward	GAAAUUGACCAGCAAUAUA
si*Stat3*	Reverse	UAUUAUGCUUGGUCAAUUUC
Ahrr	Forward	TGGACAGCACCTCTGGCTTTCT
Ahrr	Reverse	GCAAGACTGGTGCCACAATGCA
Cyp1a1	Forward	CATCACAGACAGCCTCATTGAGC
Cyp1a1	Reverse	CTCCACGAGATAGCAGTTGTGAC
Cyp1b1	Forward	GCCACTATTACGGACATCTTCGG
Cyp1b1	Forward	ACAACCTGGTCCAACTCAGCCT

Supplementary Table 3.Histological scores

Epithelial Integrity (0–4 points):
0: No damage
1: Mild epithelial injury
2: Moderate epithelial injury (loss of some epithelium)
3: Severe epithelial injury (extensive epithelium loss)
4: Complete epithelial loss
Inflammation (0–4 points):
0: No inflammation
1: Mild inflammation (few cells)
2: Moderate inflammation (localized)
3: Severe inflammation (diffuse but not full thickness)
4: Very severe inflammation (full-thickness involvement)
Structural Damage (0–4 points):
0: No structural damage
1: Mild structural changes (villi shortening, mild edema)
2: Moderate structural changes (crypt loss, moderate edema)
3: Severe structural damage (crypt abscesses, significant edema)
4: Complete structural loss (no crypts, severe destruction)
